# Age and associated hypertension impair hippocampal circuitry function and memory

**DOI:** 10.1007/s11357-025-01831-2

**Published:** 2025-10-21

**Authors:** Marcia H. Ratner, Kayla M. Nist, Richard D. Wainford, David H. Farb

**Affiliations:** 1 Department of Pharmacology, Physiology and Biophysics, Boston University Chobanian & Avedisian School of Medicine, Boston, MA USA; 2 Department of Anatomy & Neurobiology, Boston University Chobanian & Avedisian School of Medicine, Boston, MA USA; 3 Division of Cardiology, Department of Medicine, Emory University School of Medicine, Atlanta, GA USA; 4 Center for Systems Neuroscience at Boston University, Boston, MA, USA

**Keywords:** Aging, Hypertension sharp wave ripples, Cognition, Hippocampal trisynaptic circuits

## Abstract

Chronic hypertension with aging induces significant alterations in the structure and function of brain parenchyma and memory dysfunction. While therapeutic control of hypertension reduces risk, the functional changes in neural circuitry that underlie memory deficits are unknown. Identifying possible early onset changes in the hippocampal trisynaptic circuit (HTC) may reveal opportunities for therapeutic intervention. A large-scale clinical study revealed a direct relationship between reduced prospective memory score and reduced hippocampal functional connectivity in subjects with a history of chronic hypertension. Here, we report a potential mechanistic linkage between hypertension/aging and hippocampal neural circuitry dysfunction. We used chronically implanted multi-electrode silicon probes in awake freely behaving rats to monitor synchronous high frequency oscillations (at 140–200 Hz or the “ripple band”) in the HTC CA1 subregion. This hippocampal subregion is known to participate in memory consolidation, in part via HTC ripples. The nootropic drug, α5IA, that inhibits α5GABA-A receptors, was administered orally, under conditions for target engagement, and ripple amplitude measured within subject in awake resting Sprague–Dawley (SD) rats. Significant HTC and memory dysfunction was observed in aged-hypertensive SD rats. The results reveal an hypertension/age-associated anomaly in ripple band activity in SD males, characterized by reductions in mean peak ripple amplitude and in the ability of α5IA to potentiate peak ripple amplitude, without effect upon ripple frequency or duration. Thus, the population of large peak amplitude sharp wave ripples is reduced and becomes insensitive to potentiation by α5IA. Having identified a specific change in peak ripple amplitude that is resistant to potentiation by α5IA, a nootropic probe-drug, this finding could lead to nuanced circuitry level approach for the treatment of memory dysfunction with aging and hypertension.

**Supplementary Information:**

The online version contains supplementary material available at 10.1007/s11357-025-01831-2.

## Introduction

The risk for developing hypertension increases with age in humans [6]. Therefore, studying preclinical models of age-related hypertension has important translational relevance in the field of geroscience [36]. Untreated hypertension can lead to memory decline due to damaged brain vasculature, which can reduce blood flow and promote atrophy of nervous tissue, ultimately leading to vascular dementia and exacerbation of Alzheimer’s-related dementias [25, 29, 40]. Reducing mean arterial blood pressure (MAP) with antihypertensives and lifestyle changes can impede the onset of memory dysfunction [1, 10] but dementia due to neurodegeneration progresses nonetheless [11]. These clinical findings point to the significance of developing precision medicine approaches to the associated memory dysfunction.

A key large-scale clinical finding (502,537 participants) reveals a linear relationship between reduced prospective memory score and reduced hippocampal functional connectivity in participants with a history of hypertension over a wide range of blood pressures [15]. Significant associations exist between a history of hypertension and prospective long-term episodic memory and short-term numeric memory on the digit span test. Neuroimaging studies in human subjects during periods of quiet wakefulness indicate that hippocampal functional connectivity predicts the capacity to freely recall recently learned information [45]. These results in human subjects raise the concern that compromised hippocampal functional integrity might include hippocampal trisynaptic circuit (HTC) dysfunction and prompt us to explore, in this report, the use of a parallel animal model for hippocampal circuitry function in aging with hypertension.

While clinical findings have established correlative risk factors for memory impairments, the question of whether altered HTC function in vivo may underlie memory deficits has, to the best of our knowledge, not previously been reported. Age and/or hypertension may alter synaptic transmission through any number of mechanisms, whether pre- or post-synaptic, and thereby adversely affect HTC function. While it is known that the significant adverse effects of chronic untreated age-related hypertension on brain structures, including the hippocampus, can be averted by various therapeutic approaches to control hypertension, the functional engagement of the HTC in this linkage remains unresolved. Whatever the source of the memory deficits, identifying the functional changes that can be targeted pharmacologically independent of hypertension would be a crucial first step to asking mechanistic questions.

The male SD rat develops increased mean arterial pressure (MAP) and impairments in memory with aging, making it a suitable animal model for probing the role of hypertension in the onset of amnestic mild-cognitive impairment (aMCI) [13, 17, 19, 21, 26]. A hallmark of HTC function is the synchronous high-frequency oscillations between 140 and 200 Hz, also known as the ripple band due to its characteristic high-frequency wave forms that appear as ripples [5, 31]. We monitor ripple band activity during periods of quiet wakefulness using SD rats as a model for age-associated hypertension using a within-subject design. We test HTC integrity by probing circuitry function using *α*5IA, a nootropic drug that negatively modulates the action of gamma-aminobutyric acid (GABA) at α5GABA-A receptors. Synchronous high-frequency activity in the form of sharp wave ripples (SPW-Rs) recorded from the CA1 hippocampal subregion is an established functional correlate of memory replay and consolidation during sleep and awake immobility in rodents, non-human primates, and humans (Buzsáki et al., 1992 & 2015; [20, 23]). SPW-R-associated inhibitory postsynaptic currents (IPSCs), which are usually dependent on the inhibitory transmitter, GABA, are phase-locked with ripple cycles, indicating that phasic GABAergic synaptic transmission plays a role in the modulation of SPW-R oscillations [18]. α5GABA-A receptors are located extra-synaptically where they mediate tonic inhibition but are also located postsynaptically where they participate in phasic inhibition in the HTC, ripple dynamics, and memory [37]. Based on this mechanism, we describe experiments to probe the HTC with *α*5IA to ask whether ripple dynamics are altered by age and hypertension.

We report that *α*5IA increases peak ripple amplitude in young male SD rats. In addition, age and/or the associated hypertension in SD males is associated with a decrease in ripple amplitude and reduced cognitive function. We have shown previously that aging does not necessarily incur HTC dysfunction as revealed by persistent *α*5IA positive modulation of ripple amplitude [31], Fig. 5F). These results suggest that the neural network engaged in memory replay and consolidation is adversely affected by age-related hypertension, but not aging alone, but does not demonstrate causality between hypertension and HTC dysfunction.

## Methods

### Subjects

Seven male Sprague–Dawley (SD) rats (Envigo, Indianapolis, IN, USA) were used in the within-subject in vivo electrophysiological experiments. Because female SD rats do not develop age-related hypertension or memory impairments under the conditions of these experiments, only male rats were included here as a first step. Additional groups of unimplanted male SD rats (*n* = 6/group) aged 3 and 16 months were used for behavioral tests and blood pressure measurements. All rats were individually housed in a climate-controlled vivarium maintained on a regular 12-h/12-h light/dark cycle in the Laboratory Animal Science Center at the Boston University Chobanian & Avedisian School of Medicine. Rats used for in vivo electrophysiological studies had ad libitum access food and water. Rats were fed a standard irradiated normal salt rodent diet across the lifespan (Teklad Global Diet, Envigo, IN, Teklad Global 18% Protein rodent diet #2918, 18% protein, 5% crude fat, 5% fiber, total NaCl content 0.6% [102 mEq Na^+^/kg]) [17]. Rodent housing and research were both conducted in strict accordance with the NIH Guide for the Care and Use of Laboratory Animals. Boston University is accredited by the Association for Assessment and Accreditation of Laboratory Animal Care. The Boston University Institutional Animal Care and Use Committee approved all procedures described in this study (BU IACUC protocol PROTO201900015).

### Femoral artery cannulation for mean arterial pressure (MAP) recordings

Blood pressures were measured using a PE-50 cannula placed in the left femoral artery to record heart rate and MAP in separate groups of unimplanted 3-month-old and 16-month-old male rats (*n* = 6 per group). These animals were anesthetized using sodium methohexital (20 mg/kg intraperitoneally (i.p.), with 10 mg/kg administered intravenously (i.v.) as needed). Cannulation of the femoral artery and vein was performed as previously described [16], Puleo et al. 2020, [17]. An incision was made in the left femoral triangle, and the femoral artery and vein were dissected from the adjacent tissue. Using a cannula made from PE-50 tubing, the cannula was inserted into the femoral vein to allow for administration of i.v. anesthesia and isotonic saline during recovery periods. A PE-50 cannula was placed in the left femoral artery to record heart rate and MAP. Cannulas were tied in place using sutures, and the incision was closed. Rats were positioned in a Plexiglas rat holder, and the cannula for the femoral artery was attached to an external pressure transducer, while the cannula for the femoral vein was attached to an infusion pump. A 2-h recovery period was allowed, during which an i.v. infusion of isotonic saline (20 μL/min) was performed, and rats returned to full consciousness, with stable cardiovascular and renal function. After the 2-h recovery period, baseline MAP was continuously recorded over a 1-h period in conscious rats through the femoral artery cannula using the computer-driven data acquisition software, MP150 and Acknowledge 3.8.2 (BIOPAC, CA).

### Tail-cuff blood pressure measurements

Blood pressures in the aged male SD rats (*n* = 4) used in the in vivo electrophysiology studies were measured non-invasively using the tail-cuff method. Blood pressure was measured prior to cranial implant surgery using a WPI MRBP system (World Precision Instruments, Sarasota, FL, USA). The in vivo electrophysiologist was blinded to the results of these measurements, which were not correlated with the results from neurophysiological testing until after all the data had been acquired and processed.

### Cranial surgery for microelectrode array placement

Rats were anesthetized with isoflurane (3.5% for induction; 1.5–2% thereafter) in 100% oxygen delivered via a calibrated vaporizer (Vaporizer Sales & Services Inc., Rockmart, GA, USA). The heads of the animals were shaved and prepped for surgery with betadine in triplicate. After placing the rats in a stereotaxic instrument (David Kopf Instruments, Tujunga, CA, USA), a midline sagittal incision was made across the top of the head to expose the cranium. A small craniotomy (~ 2 mm in diameter) was created immediately above the right dorsal hippocampus with a center located at 3.6 mm AP and 2.5 mm ML from Bregma. The dura mater was excised with a dura knife, and the silicon microelectrode array was lowered into position above the surface of the neocortex. Ten additional holes (< 0.5 mm) were drilled into the skull for the placement of an equal number of stainless-steel screws; two of these screws served as electrical grounds as well as providing additional anchoring points for securing the movable microelectrode array and copper mesh Faraday cage to the skull with dental acrylic (Patterson Dental Supply, St. Paul, MN, USA) [41]. The use of the Faraday cage in these experiments reduces electrical and radio frequency artifacts [41],Liu et al., 2023). At the end of surgery, the silicon microelectrode array (NeuroNexus, Inc. Ann Arbor, MI, USA) was connected to an Intan RHD system (Intan, Inc.) for positioning of the probe within the CA1 subregion based on real-time neural input. Electrode placement within the CA1 subregion was also adjusted after recovery from surgery and confirmed daily prior to training and test sessions by inspection of the local field potential (LFP) for evidence of activity during periods of ambulation and ripples during periods of immobility [3–5]. These two neural activity metrics are well-established functional real-time indicators of electrode localization within the CA1 hippocampal subregion [3–5]. Single shank silicon probes with linear electrode arrays that span across CA1 layers (str. oriens, pyramidal layer, and str. radiatum) were also used in two of the rats included in these experiments to ensure that SPW-Rs are confidently measured and to facilitate rejection of artifacts due to chewing and movement [23] (see Supplemental data section Figs S1-S4).

### Oral drug administration

The GABA-A *α* 5-selective positive allosteric modulator *α* 5IA (3-(5-methylisoxazol-3-yl)−6-[(1-methyl-1,2,3-triazol-4-yl) methyloxy]−1,2,4-triazolo[3,4-a] phthalazine) (Sigma-Aldrich Inc., USA and Tecoland, Irvine, CA, USA) was chosen based on its unique pharmacodynamic profile, oral bioavailability, and our previous results demonstrating that it dose-dependently increases ripple band power and peak ripple amplitude in rats [31]. The vehicle (0.5% 400 centipoise methylcellulose) without or with drug was administered via oral gavage 30 min before each test session. To facilitate oral administration, rats were mildly sedated by briefly placing them in an induction chamber filled with 5% isoflurane. The drug was always administered after the vehicle; counterbalancing the order of vehicle and drug administration was not possible due to the duration of the experimental protocol, the plasma half-life (0.9 h) of the drug, and the within-subject study design used for testing responsivity to *α* 5IA.

### Escalating cumulative dose paradigm for analysis of ripples

These experiments used our established probe drug challenge model, which combines escalating doses of *α* 5IA with serial recordings of LFPs from the CA1 subregion, while the animals are awake and immobile in a familiar environment [31]. This method was selected because different behaviors, such as running, sleeping, and wakeful resting, influence the occurrence of ripples in the CA1 hippocampal subregion. During the training phase, rats were allowed to explore a black plywood square (60 cm × 60 cm) enclosure with vertical yellow stripes on one of the four walls for at least 7 days to establish this as a “familiar environment.” Serial 10-min recordings were made while rats were immobile, resting quietly in this familiar environment. Because electrode depth can influence power in the ripple band, the electrodes were not moved on the day of testing. For each series of experiments, we used single electrodes on each shank with optimal positioning in the region of interest to optimize comparisons within subjects.

On probe drug testing day, the subject receives an oral dose of vehicle or drug in the escalating dose series. Vehicle is administered at 30 min prior to the first recording session. The subject is returned to the home cage for 30 min after dosing with vehicle or escalating doses of probe drug to permit for absorption and distribution. At the end of the 30-min interval, the animal is then placed in the familiar environment (F). Recording is initiated when exploratory ambulation ceases, judged by sustained immobility while remaining awake and resting quietly. At the end of the 10-min recording session, the subject is administered the next higher dose in the series and returned to the home cage for 30 min to allow for drug absorption prior to the next recording session. This process is repeated for each dose in the escalating dose series. The environment is cleaned with 70% ethanol solution between sessions to remove odors. The standard escalating dose protocol is vehicle F1, dose-1 F2, dose-2 F3, and dose-3 F4, where the dose listed in each figure represents the cumulative dose of drug at the time of each measurement. The same environment F is used in all 4 recording sessions, where F1 is the first exposure of the subject to the familiar environment and F4 is the 4th exposure to the familiar environment. All escalating doses of drug were performed in the afternoon. All subjects experienced the same experimental protocol, with each rat serving as their own within-subject control using vehicle. It is recognized that exposure to isoflurane transiently reduces MAP [46]. Recordings were made 30 min after a very brief period (2–3 min induction time) of anesthesia to sedate implanted rats sufficiently to facilitate handling during oral gavage dosing with probe drug. At the time of recording, all rats were fully ambulatory and showed no overt signs of residual sequelae due to this brief period of anesthesia. This observation is consistent with a recovery time of 4.1 ± 1.2 min following a brief surgical procedure in rats [2].

### Data acquisition and post acquisition processing for analysis

Three of the four aged SD rats were implanted with B32 type four shank silicon probes for effectively targeting a single subregion such as CA1 (NeuroNexus, Inc. Ann Arbor, MI, USA). Young SD 1 and aged SD 5 were both implanted with NeuroNexus A1X32 style single long shank probes which span multiple regions (e.g., CA1 and radiatum) allowing for signal comparison and ensuring the observations seen in these investigations are not artifacts of probe position. These probes also allowed us to determine electrode locations based on the distinct oscillatory patterns observed in different hippocampal subregions such as the hippocampal fissure based on theta power and the CA1 subregion based on the presence of ripples (Buzsáki et al., 2002). These region-specific functional signatures can be effectively used for real-time prediction of electrode localization during recordings and subsequently confirmed with post hoc histology (Buzsáki et al., 2012; Sullivan et al., 2012; [31]). Neural signal data was acquired using an Intan RHD system (Intan Technologies, Los Angeles, CA, USA) and processed using NeuroExplorer.

Electric fields monitored with extracellular electrodes provide useful information about state-dependent neuronal activity. The LFPs recorded with indwelling microelectrodes represent the sum of the synchronized local changes in post-synaptic potentials measured in millivolts (mV). The greater the distance of the recording electrode from the source of these voltage changes, the less accurately the LFP reflects the activity of interest (e.g., activity in the CA1 pyramidal cell layer) (Buzsáki et al., 2012). The characteristics of the LFP signal include frequencies and amplitudes which reflect the changes in current flux associated with excitatory and inhibitory post-synaptic potentials [47]. The LFP signal can be frequency filtered to allow for studying activity within specific frequency ranges. Analysis of data from an LFP signal that has been frequency filtered for activity in the ripple band (140 to 200 Hz) provides information about the characteristics of the ripple events that give rise to both the raw LFP and the PSD plot of activity in this same frequency range. The power spectral density (PSD) plot of the LFP, which reflects the relationship between the energy of the extracellular signal and its distribution across different frequencies, is generated from the results of a fast Fourier transform of this signal. The mean and peak amplitude and frequencies of these events are reflected in the shape of the PSD plots. For this reason, we present both the raw data as a PSD plot and the computationally disambiguated peak ripple amplitude, mean ripple amplitude, ripple frequency, and ripple duration to improve the clarity of the neurophysiological findings.

Ripple event metrics were detected and characterized using the well-established method of the Buzsáki Laboratory. Briefly, the LFP data was band-pass frequency filtered for ripple band activity from 140 to 200 Hz using a Butterworth filter. An event peak threshold of 5.0 SDs of the band-filtered LFP was used to identify potential ripple oscillations. Two additional thresholds defined as greater and less than 2.0 SDs of the filtered trace were used to define the beginning and end times for each ripple event. Ripples occurring within less than 30 ms of each other were merged into a single event. Only events with durations greater than 20 ms and less than 100 ms were included in the final analyses for significant differences in the averages of the mean and peak ripple amplitudes, peak frequencies, and durations of ripple events across the recording sessions. To ensure data reproducibility and improve rigor and transparency, a commercially available program (NeuroExplorer) was used to detect and process ripple events and calculate the ripple event metrics used in these studies. This commercially available platform was selected for this purpose with the goal of increasing the translational value of ripple data acquired by different laboratories [23, 32]. The results show differences in LFP across animals as presented in Fig. 2C.

Blinding of study: The age and MAP data were recorded by a member of our collaborating lab (AM) and sealed until completion of the experiments. The electrophysiologist was blinded to age and MAP of subjects during experiments, data processing, and all within-subject analyses. The unmasked age and MAP data was only used for the post hoc between-group comparisons and for linear regression analysis.

### Behavioral testing

#### Novel object recognition test

For the Novel Object Recognition test (NORT), rats were placed in a 60 by 60 cm open-topped behavioral testing arena with a video camera fixed above to record testing for manual analysis. Two different objects were placed in separate quadrants of the arena, placed 6 in from adjacent corners. For familiarization, rats were placed in the center of the arena and allowed to explore the environment and objects for 10 min. Following familiarization, rats were returned to their home cages for 2 h. Following the 2-h latency, rats were returned to the testing arena, this time with a novel object replacing one of the familiar objects. Rats were allowed to explore the environment and objects for 10 min to complete the test portion of the assessment. After testing, rats were returned to their home cages. The testing arena and objects were thoroughly cleaned between each test with 10% ethanol.

#### Location novelty recognition test

For the location novelty recognition test (LNRT), rats were placed in a 60 by 60 cm open-topped behavioral testing arena with a video camera fixed above to record testing for manual analysis. Two different objects were placed in separate quadrants of the arena, placed 6 in from adjacent corners. For familiarization, rats were placed in the center of the arena and allowed to explore the environment and objects for 10 min. Following familiarization, rats were returned to their home cages for 2 h. Following the 2-h latency, rats were returned to the testing arena, this time with one of the familiar objects placed in the opposite quadrant from its original location. Rats were allowed to explore the environment and objects for 10 min to complete the test portion of the assessment. After testing, rats were returned to their home cages. The testing arena and objects were thoroughly cleaned between each test with 10% ethanol. The time a rat spent investigating each object separately was measured. Time investigating the novel object or location and the total time spent investigating both objects was used to calculate the discrimination index and the percent of time exploring the novel object/location. The familiarization score was calculated as the absolute value of the time spent at one object subtracted by the time spent at the other object divided by the total time spent investigating the objects. This score was used to assess any preference a rat may have for one object over the other.

$$\mathrm{familiarization}=\frac{\left(\text{time spent at right object}\right)-\left(\text{time spent at left object}\right)}{\text{time spent at both}}$$

Discrimination index is a measure of sensitivity and is assessed as the time spent investigating the novel object/location minus the time spent investigating the familiar object/location divided by the total time spent investigating both objects/locations. Percent of time exploring the novel object/location was assessed based on time spent investigating the novel object/location divided by the total amount of time spent investigating.

$$\text{discrimination index}=\frac{\left(\text{time spent at novel object or location}\right)-\left(\text{time spent at familiar object or location}\right)}{\text{total time spent at both}}$$

### Statistical analysis

The processed ripple metric data was saved as a comma separated values (.csv) file and imported into GraphPad Prism for statistical analysis. Ripple metrics analyzed included peak and mean ripple amplitudes (mV), peak ripple frequency (Hz), and ripple duration (sec). Appropriate non-parametric tests including Friedman’s ANOVA and the Kruskal–Wallis test were used for all within-subject comparisons of ripple metrics. Student’s *t* test was used for between-subject comparisons of average percent change in peak ripple amplitude in young SD versus aged hypertensive SD rats. Between-subject comparisons of ripple band power distributions were performed on *Z* score normalized data sets using the Kolmogorov–Smirnov test. Results of linear regression for % change in maximum ripple amplitudes (response variable) versus predictor variables (MAP and age) are presented with the 95% confidence intervals. An unpaired *t* test was used for comparison of performance on the object and location recognition tests.

## Results

### MAP in aged SD male rats

The MAPs of the aged male SDs were measured using a WPI MRBP tail-cuff system. The MAPs of the aged SDs included in this study ranged from 142 to 183 mmHg with a mean of 160 mmHg (see Supplemental Data Table S1).

### Blood pressures and response to α5IA probe drug challenge

Within-subject analysis of the results of in vivo electrophysiological recordings of high-frequency neural activity in the ripple band revealed rats that were positive *responders* and rats that were *non-responders* to *α*5IA probe drug challenge. Of the SD rats tested (*n* = 7), 29% did not show a positive response to probe drug challenge. Among the aged SD rats, 50% were non-responders. This change in response to probe drug challenge was associated with MAPs and SBPs greater than 160 mmHg.

### Power spectral density plots reveal changes in characteristic neural activity patterns

**Fig. 1:**
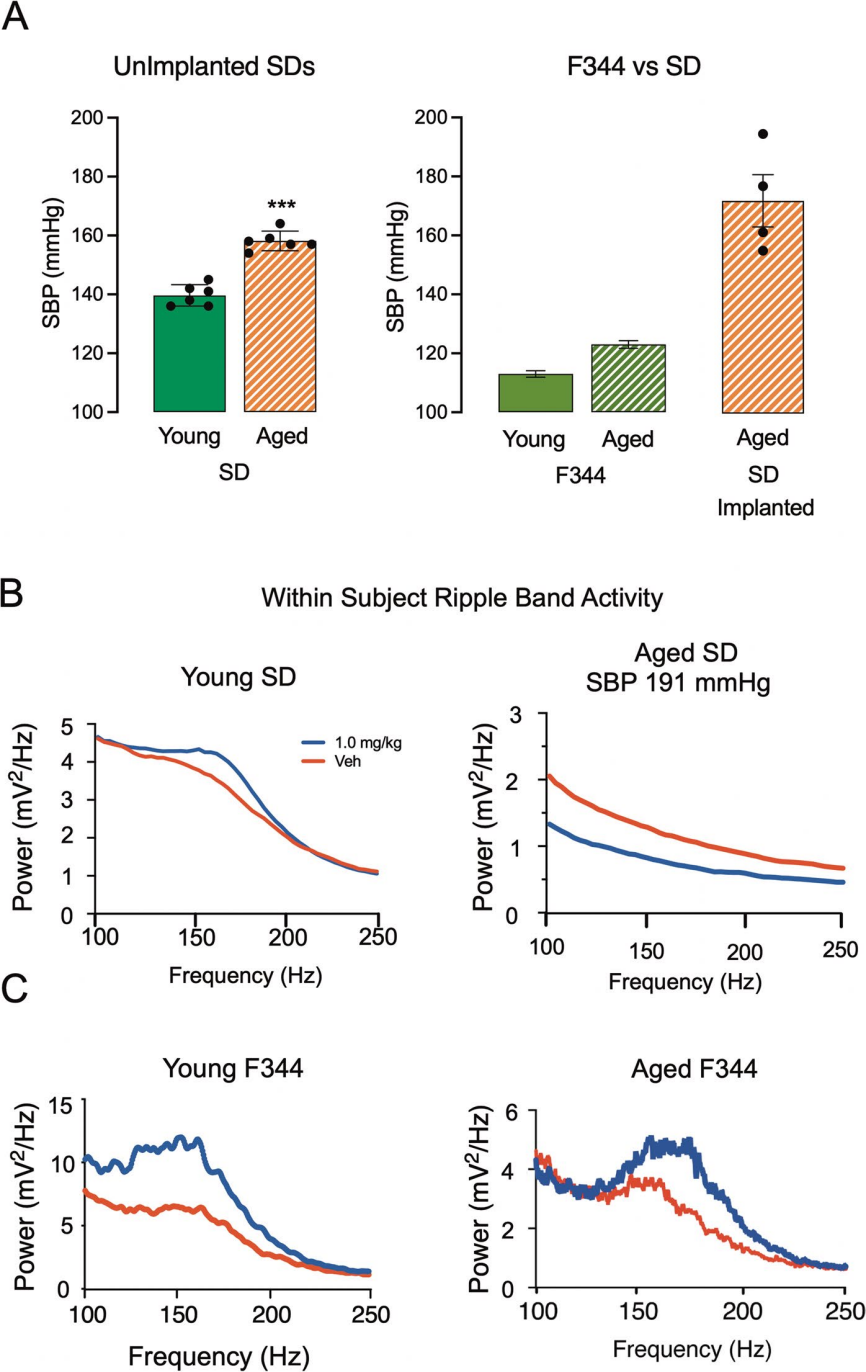
Hypertension in aged SD rats is associated with anomalous responses to α5IA probe drug potentiation of ripple band amplitude. **A** Systolic blood pressure (SBP) in groups of unimplanted young (aged 3 months) and (aged 16 months) SD males (left panel). In the right panel, SBPs in young and aged F344 males are shown for comparison with SBPs of hypertensive implanted SD males. **B** Ripple band responses to α5IA probe drug challenge in SD male rats. A young SD male shows a robust ripple band response to probe drug challenge, while an aged SD male with SBP of 191 mmHg shows an inhibited rather than potentiated ripple band power response to α5IA challenge. **C** Male F344s aged 9 and 18 months, which are not prone to age-related hypertension in this age range, show robust ripple band (140 to 200 Hz) responses to α5IA probe drug, while a TgF344-AD rat does not. All data shown are within-subject changes in ripple band power at each frequency from 100 to 250 Hz following administration of vehicle (red) or 1.0 mg/kg α5IA (blue). Blood pressure data are means ± SEMs. Significance in panels A and B is indicated by *** at *p* < 0.001. Unimplanted F344 SBP data are derived from [30], which were replicated and extended to show that MAP also does not significantly increase with aging in the TgF344-AD animals, see Fang 2023 [14]. **C** Implanted F344 and TgF344-AD power spectral density plots are reproduced from Ratner et al. [31] for comparison with SD spectra

The Fischer 344 (F344) is the most frequently used rat model in studies looking at the effects of aging. Unlike male SDs, which are hypertensive by 16 months, male F344 rats do not show evidence of significant high blood pressure at ages up to 18 months [30] (Fig. 1A). The results of the current studies using our established model for testing HTC integrity based on the ripple band (140 to 200 Hz) response to probe drug challenge with *α*5IA revealed a positive response in young SD males and a loss of the response in aged SD males with systolic blood pressures greater than 160 mmHg (see Fig. 1B). The results of our previous experiments using this same Fischer rat model have revealed a positive ripple band response to α5IA probe drug challenges in male F344s up to the age of 18 months [31] (see Fig. 1C).

**Fig. 2:**
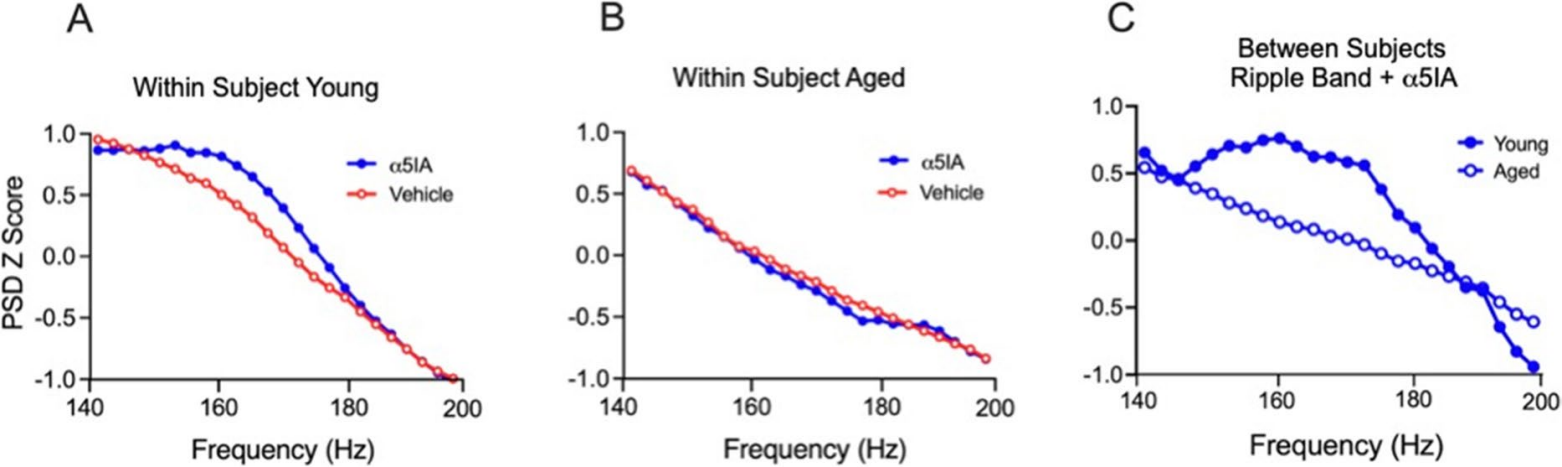
Age-associated high MAP disrupts the ripple band. Power spectral density (PSD) *Z* score normalized plots on the ordinate axis with frequencies in Hz on the abscissa following administration of vehicle and 1.0 mg/kg of α5IA probe drug. **A** Within-subject PSD *Z* score plot distribution of a young SD rat showing an increase in ripple band power in response to α5IA probe drug challenge with an overt hump in the ripple band. **B** Within-subject PSD *Z* score plot from an aged high MAP SD (MAP 183 mmHg) reveals a conspicuous absence of the characteristic ripple band hump in the response to α5IA probe drug challenge. **C** Between-subject *Z* scored PSD plot distributions of the merged 1.0 mg/kg data from the young rats (*n* = 3) and aged SDs (*n* = 4) reveal a significant difference in the distributions in response to probe drug challenge in young and aged SDs (Kolmogorov–Smirnov, *p* = 0.002)

To further evaluate the effects of blood pressure on within and between subject ripple band activity responses to probe drug challenge, the *Z* score plots of ripple band power distribution from 140 to 200 Hz in young and aged rats were analyzed based on MAP values (see supplemental Table S1) which, because it includes diastolic pressure, is considered a more accurate indicator of organ perfusion. The results of these analyses revealed that age-related increases in MAP are also associated with a conspicuous absence of the well-recognized characteristic hump in the ripple band [5, 31, 39] in the aged SDs as compared with young SDs (Fig. 2A and B). Analysis of the merged 1.0 mg/kg data from the young SDs (*n* = 3) and aged SDs (*n* = 4) reveals a significant difference between these groups in the response to *α*5IA probe drug challenge (Fig. 2C).

### Relationship between peak ripple amplitudes and age and/or MAPs

**Fig. 3:**
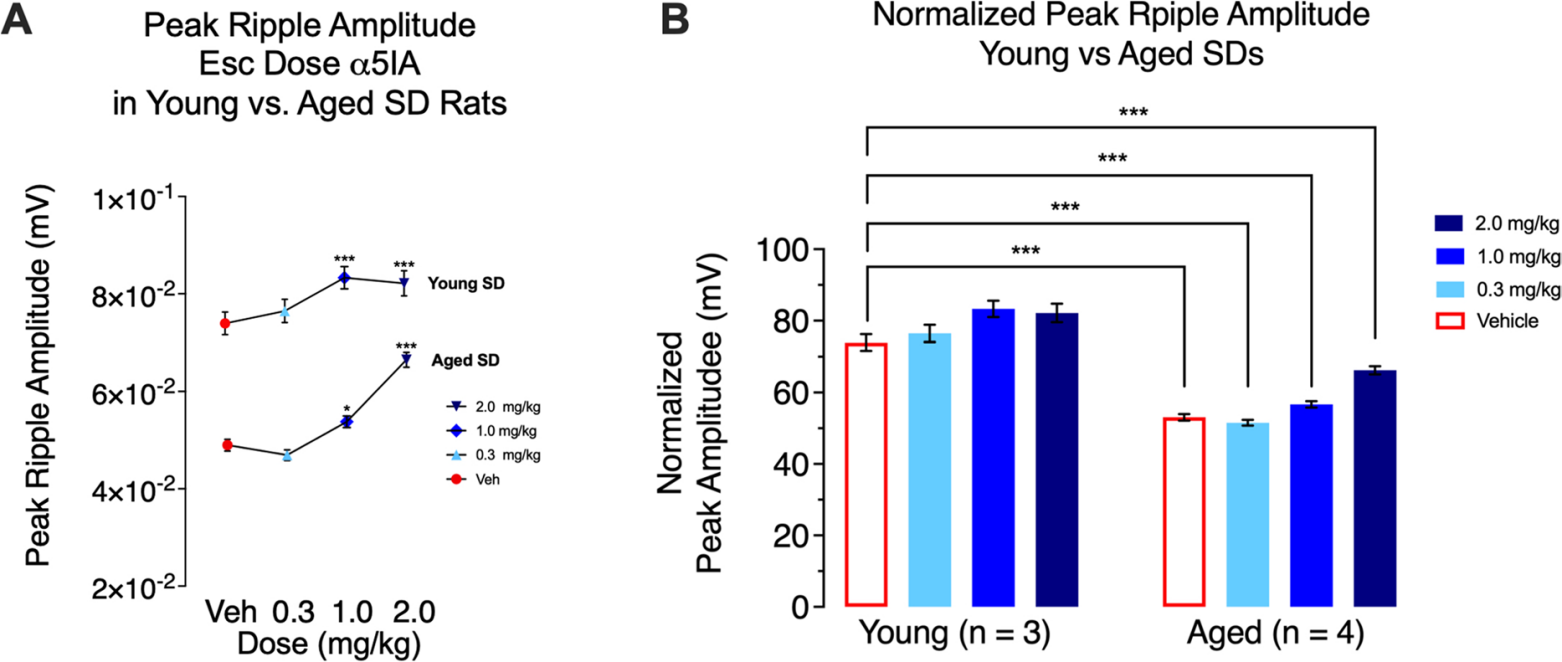
Comparisons of peak ripple amplitudes in young and aged SDs. **A** Results of within-subject analysis for young (*n* = 3) and aged (*n* = 4) SDs indicate that peak ripple amplitudes are significantly (*p* < 0.001) increased in both groups. **B** Results of between-subject analysis of normalized data indicate that peak ripple amplitudes are lower in aged SDs at all doses tested. Significance indicated by * at *p* < 0.05 (see * over 1.0 mg/kg aged SD value vs. veh); *** at *p* < 0.001 (all other comparisons in **A** and **B**)

Peak ripple amplitudes, which were previously shown to be sensitive to Alzheimer’s disease (AD) neuropathology in the TgF344-ADs, were then analyzed both within-subject and between-group differences in the SDs. Although the aged SDs did respond to *α*5IA probe drug challenge, the between-group analyses of the data from young and aged SDs revealed substantially lower peak ripple amplitudes in the aged SDs at all doses tested (Fig. 3).

**Fig. 4:**
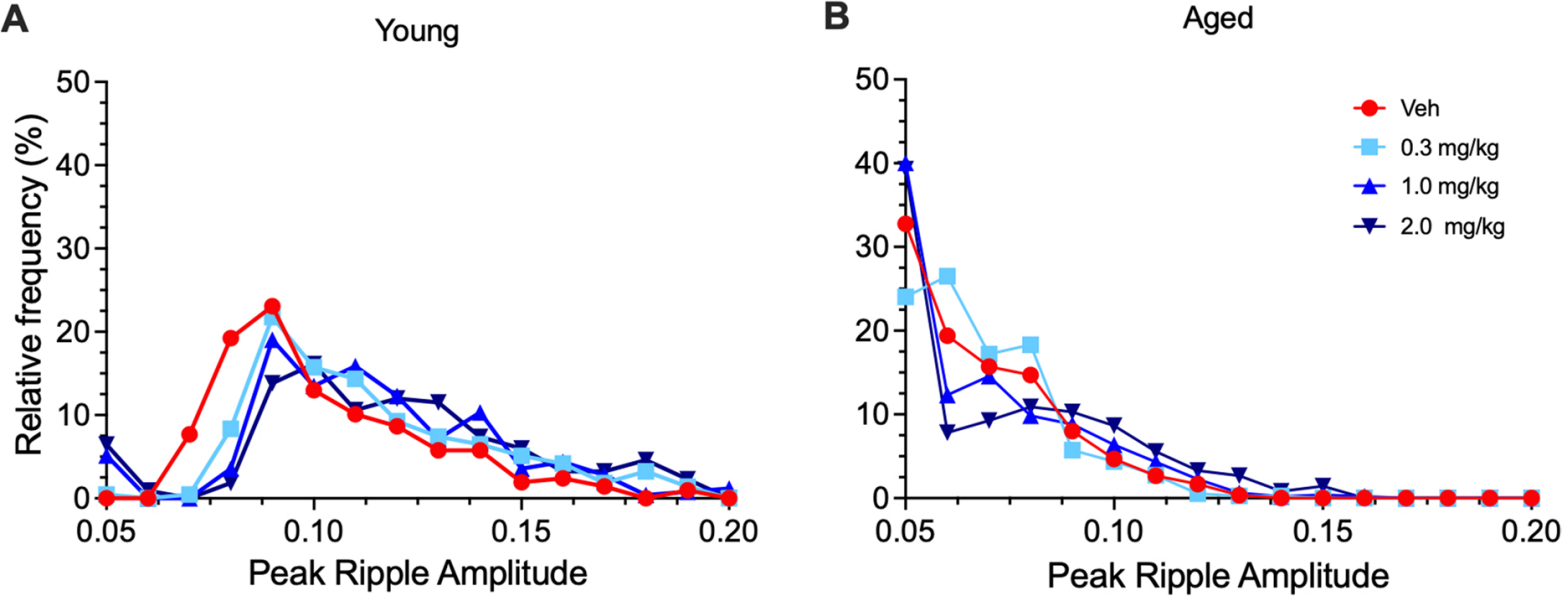
Peak ripple amplitude frequency distributions of aged SD rats are shifted to the left. **A** The average relative peak ripple amplitude frequency distributions for the three young SD rats following escalating doses of α5IA show a drug-induced shift to the right with progressively higher doses of probe drug. **B** The average relative frequency distribution for peak ripple amplitudes in the four aged SDs following administration of α5IA shows a distribution that favors lower amplitude events by comparison with the young SDs. Key shown in panel **B**

Next, we compared the relative frequency distributions of peak ripple amplitudes in young vs. aged SD rats at escalating doses of *α*5IA (within-subject) and observed a left shift with age, meaning that the population of ripples with low amplitude increases with age (Fig. 4). *α*5IA shows a right shift in ripple amplitude from vehicle (red determinations on left) to drug *α*5IA (blue points from dose escalation experiments withing subject all shifted to the right) in three young but not four aged hypertensive SDs. This indicates that in aged SD animals, the large amplitude ripples that remain are nonresponsive to modulation by *α*5IA.

**Fig. 5:**
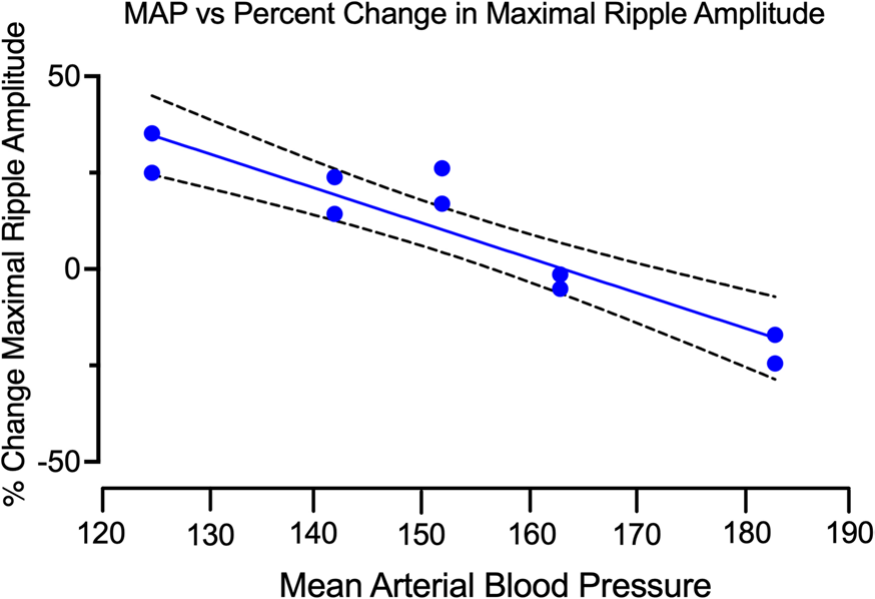
Peak ripple amplitude is inversely correlated with increasing blood pressure. Linear regression analysis of % change in the maximum ripple amplitude (*y*-axis) versus mean arterial blood pressure (*x*-axis). Results of linear regression using data from 14 experiments on 7 SDs in two age groups, young and aged, indicate R^*2*^ = 0.86; *F* = 48.74; DFn, DFd = 1, 8; *p* < 0.01; a total of 14 maximal drug response determinations. The first two points were from 3 young normotensive SDs comprised of maximal responses to 1 and 2 mg/kg α5IA at each dose that were not significantly different from each other. The 3 determinations at each of the two doses were also not significantly different from each other, as predicted if the experiment had determined the maximal response to drug in vivo. The average of the data from each of the two doses is thus shown for clarity. The 1.0 and 2.0 mg/kg doses of α5IA are used to establish the maximal potentiation of peak ripple amplitude in agreement with the established maximal receptor occupancy in vivo in rat.

To assess for a relationship between the predictor variable (MAP) and the response variable (peak ripple amplitude) at full GABA receptor occupancy, we examined the % change in maximum ripple amplitude following administration of the 1.0 and 2.0 mg/kg doses of *α*5IA vs. MAP. The results in Fig. 5 indicate that % change in peak ripple amplitude decreases with increasing MAP (*R*^2^ = 0.86; *p* < 0.01) (Fig. 5). Taken together, the results are consistent with an apparent continuum of responsivity to *α*5IA at doses expected a priori to produce maximum receptor occupancy that is negatively correlated with increasing MAP (Atack et al., 2009; [31]).

### Relationship between behavioral test performance and MAP

**Fig. 6:**
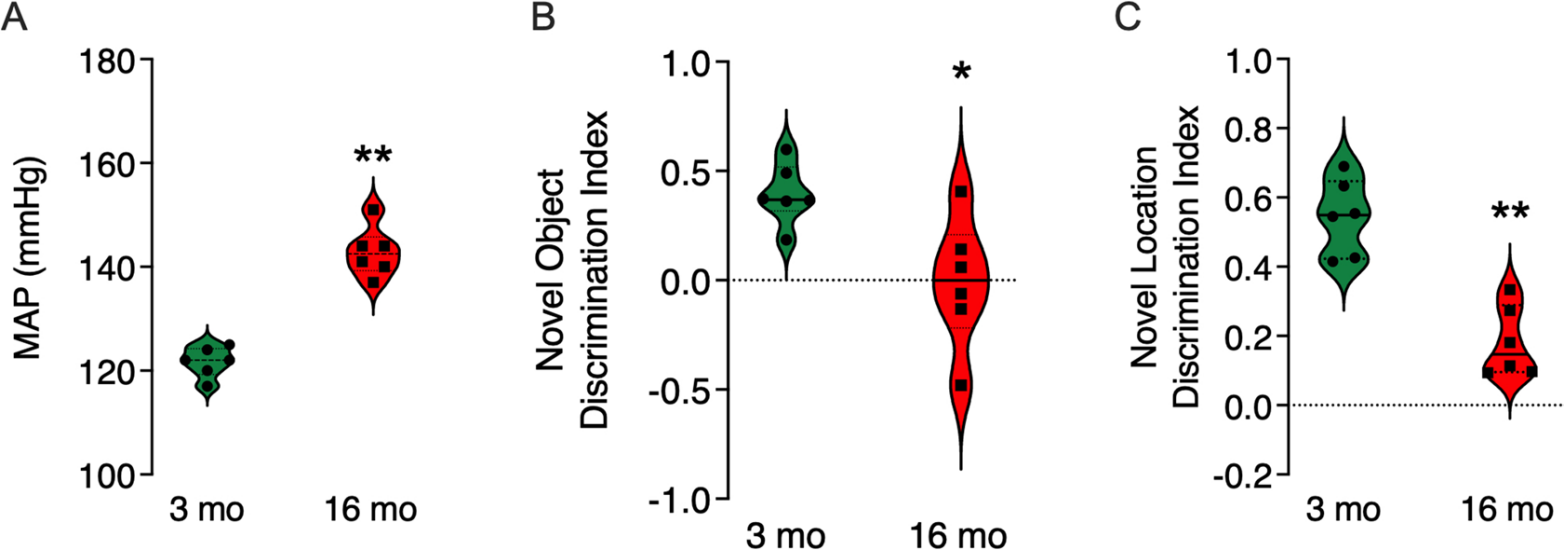
Novel object recognition test and location novelty recognition test results for 3-month and 16-month male SD rats. **A** Invasive measurements of MAPs via femoral artery confirmed hypertension is seen in 16-month SDs but not in 3-month SDs. **B** Novel object discrimination index scores for 3- and 16-month male rats reveal a significant difference between groups based on age at testing. **C** Novel location discrimination index scores for 3- and 16-month male rats also reveal a significant difference between groups based on age at testing. Results shown are from unpaired *t*-tests. Significance indicated by * at *p* < 0.05 and ** at *p* < 0.01

We performed blood pressure measurements of MAP and basic behavioral testing in separate groups of male SD rats. The results of these studies extended and confirmed previous observations of increased MAP with aging in 16-month SDs (Fig. 6A). Performance on the delayed recall test sessions of both the NORT and LNRT revealed memory performance deficits consistent with the in vivo electrophysiological observations reported on herein (Fig. 6B and C) (see supplemental data section, Fig. S5).

## Discussion

The objective of this research was to search for signaling in the hippocampus that would correlate with or be predictive of memory dysfunction associated with aging-associated chronic hypertension. While the results cannot differentiate between this hypertension and aging at this time, they represent, to the best of our knowledge, the first demonstration of a mechanistic linkage on the neural circuitry level between HTC dysfunction in age-associated hypertension. While the extent to which these preclinical results will effectively translate to human geroscience remains to be seen, recent findings in humans using non-invasive technologies such as magnetoencephalography to measure changes in ripple band activity suggest that translation to humans may be feasible [38, 43].

A direct relationship between a reduced prospective memory score and reduced hippocampal functional connectivity in subjects with a history of hypertension over a wide range of blood pressure was observed in a large-scale clinical study [Feng 2020]. We hypothesized that reduced functional connectivity due to hypertension might disrupt high frequency oscillations, as measured in local field potentials (140–200 Hz) in CA1. These high frequency oscillations or “hippocampal ripples” are generally accepted as a biomarker of memory function in both humans and animal models of memory mechanisms investigations [23]. Ripples can be thought of as waves or packets of historical information that have been newly acquired, recalled, and consolidated or reconsolidated [9]. We focused on a characteristic oscillation referred to as a sharp wave ripple (SWR) due to its shape and foundational support for a role in the mechanism of memory consolidation (Buzsaki 2015).

As a first step toward addressing causality as compared with concordance of the effects of age-related hypertension on memory, we employed high-density in vivo silicon probe electrophysiology to record from the CA1 subregion of the hippocampal HTC to establish baseline ripple band parameters (ripple amplitude, frequency, and duration). The results compare young to aged SD rats and reveal differences in high-frequency oscillations in the ripple band associated with age-related increases in MAP. Aged male SD rats are known to develop increased blood pressure and impairments in cognitive function as assessed by the NORT and LNRT tests [17, 26].

Between subject comparisons of within subject determinations show that aged hypertensive SD rats have lower baseline peak ripple amplitudes. But does the severity of age-related hypertension increase HTC dysfunction? Between-subject analysis of aged SDs stratified by MAP reveals significant between-group differences at all doses of α5IA tested, with the exception of 2.0 mg/kg α5IA in MAP < 160 mmHg SDs (see supplemental Table S2). Linear regression analysis shows that % change in maximum ripple amplitude decreases with increasing MAP (Fig. 5). By stratifying SD rats based on response to α5IA and MAP, differences in ripple band activity were uncovered by using the same subject as its control. A much larger data set involving many more chronically instrumented animals would have been required to resolve this difference if subjects were treated as a uniform group. The within-subject MAP-dependent changes include (1) the loss of a distinctive hump in the ripple band (which is characteristic of healthy young adult rat HTC function); (2) a shift of ripple amplitude distribution to lower amplitude oscillations; (3) a progressive decrease in the ability of α5IA to potentiate ripple amplitude; and (4) an apparent loss of the ability of α5IA to potentiate ripple amplitude with MAP ≥ 160 mmHg.

These findings, when taken together, are consistent with but do not demonstrate that untreated age associated increased MAP disrupts functional hippocampal circuitry. Consistent with this interpretation, impaired performance on the delayed recognition trial of the NORT and LNRT was related to age associated increased MAP measured in a separate group of aged male SD rats. These behavioral findings are consistent with those previously reported in other rat models of hypertension and aging [13, 19, 21]. Additionally, we found that age-associated increases in MAP disrupt high frequency neural network oscillations in the ripple band. Ripples have been linked to memory replay and consolidation (reviewed in [5]).

The results of the α5IA modulation experiments support the hypothesis that tonic and/or phasic inhibitory control of CA1 ripple architecture results in increased peak ripple amplitude in young adult SD animals but is disrupted in SD rats with MAP greater than 160 mmHg. The severity of hypertension in the group of four aged subjects correlates with greater functional deficits. Interestingly, analyses of ripple event metrics from aged SD rats, which did not show the characteristic ripple band hump, nevertheless displayed a positive response to the probe drug challenge with significant increases in peak and mean ripple amplitudes. This suggests that rats with less severe age-associated increased MAP may initially retain some functionality, or the neural circuitry can compensate for the early adverse effects of hypertension. These in vivo electrophysiological findings in aged increased MAP SD rats are consistent with the hypothesis that untreated hypertension may lead to age-related neural network dysfunction implicated in aMCI and Alzheimer’s disease (AD) [8, 12, 14, 22, 24, 28, 33].

CA3 population bursts, which give rise to ripples, are shaped by activity patterns in the dentate gyrus and entorhinal cortex, increasing the likelihood of ripples when dentate gamma power (50–100 Hz) is optimal [39]. These observations, combined with our findings of functional changes in the ripple amplitude distributions shifting to lower amplitude with age-associated increased MAP, suggest that upstream dysfunction in the entorhinal cortex, dentate gyrus, CA3 subregions, or some combination of them will almost certainly play a role in the qualitative differences observed in the PSD plots and the quantitative modulatory responses to α5IA. It is noteworthy that studies of 6-month-old spontaneously hypertensive Wistar rats have shown a reduction in grey matter volume in both the dentate gyrus and CA1 subregions, alongside a loss of neurons in the CA1 subregion [34, 35].

What is the potential impact of untreated BP and of anti-hypertensive medications on neural circuits engaged in memory? This study has several limitations and strengths.

### Limitations and future directions

1. We did not record neural data from other hippocampal subregions, such as the dentate gyrus (DG), so we cannot determine whether changes in DG gamma power contribute to the observed changes in high frequency LFPs.
2. All recordings reported were made continuously from an optimally positioned single electrode (out of 32) located in CA1 throughout an experimental series. Thus, a strength of the approach is to focus on a finite component of the local circuitry involved in memory. However, at the same time, this precluded measuring MAP immediately prior to or after recordings due to the risk of shifting the implant. Therefore, MAPs were measured before rats were implanted and might not be unaltered at the time of each recording.
3. The behavioral aspects of this study are limited to the interactions between probe drug administration and periods of spontaneous immobility when theta power is low and ripple events are more likely to occur. Therefore, the influence of α5IA on neural activity patterns associated with ambulation, such as theta-gamma coupling, could not be effectively investigated (van den [42].
4. The rats used in these studies were purchased from Envigo, so it is unknown if male SD rats purchased from other vendors will also develop age-dependent hypertension over the same time frame [17].
5. Hypertension exhibits systemic effects on various brain regions, each of which may contribute directly or indirectly to HTC function. Thus, we will attempt to determine causality in future experiments with several novel approaches. Nevertheless, these pitfalls do not diminish the importance of establishing for the first time that HTC dysfunction is associated with aging-associated hypertension and memory dysfunction.
6. Previous studies have demonstrated that brief periods of rest or “wakeful resting” facilitate ripple generation and consolidation of new memories, indicating that sleep is not an absolute requirement for investigating the linkages between ripple modulation and memory function [44]. That said, a limitation of within-subject tests of ripple function in response to probe drug with the awake immobility (wakeful resting) model is that the neural data recorded while these animals are awake does not allow for a correlation with exposure to any specific episodic event(s). However, it has been shown that rats remember the trial-unique stream of multiple events (episodes) and the order in which these events occur, such as the serial exposures separated by time between doses used in our FFFF model. This aspect of memory function has also been shown to be mediated by engaging hippocampal-dependent episodic memory processes [27].
7. The complexity of establishing stable long-term recordings from an optimized electrode limits the number of subjects for the study, which we addressed in part by using a within-subject method where each animal serves as its own control. Nevertheless, sample size is limited, with groups of three young and four aged rats. We have replicated the potentiation of ripples in young adult and aged animals in three strains of rats: Long Evans, F344, and now young adult SD for a total of 7 rats. However, we have not yet expanded this study to other models for hypertension or added more animals to the existing cohort. We plan to do this as part of future research. For now, we acknowledge the significant challenges while addressing aging, blood pressure, and memory as variables. We plan to address these issues in future studies.

An age-related loss of ripple band power and a5IA potentiation is not a characteristic of aging rats. For example, F344 male rats are not prone to developing age-related hypertension [7, 30] and the within-subject ripple band response to α5IA probe drug persists up to 18 months of age in F344 males [31]. This observation suggests that aging alone may not be associated with disruption of HTC function. Of course, we can only see what we can monitor, and further research will be needed to assess whether hypertension unrelated to aging causes HTC dysfunction or a distinct dysfunction in aging SD rats is at play.

8) This research is based on the hypothesis that receptor subtype preferring, or selective pharmacological modulators, can be used to assess neural circuitry function. However, as stated in limitation 1 above, we did not record neural data from other hippocampal subregions or upstream circuits, such as the dentate gyrus (DG), so we cannot determine the source(s) of the altered local field potentials. Future research should focus on determining GABAergic tone, synaptic transmission properties, and upstream circuitry activity that combines electrophysiology with histological analyses to assess whether HTC dysfunction is at play or is reflecting upstream dysfunction(s).
9) The behavioral results were included to determine whether aging and hypertension in our hands did affect memory. However, novel object recognition and location novelty recognition do not assess specific domains of memory that select for hippocampal trisynaptic integrity. The sample size was small, and the results could drift if larger groups of rats were tested. We did not pursue this because our results were confirmatory of published data [13, 19, 21]. Behavioral analyses, while showing significant differences between young and aged groups, are limited in scope, do not add independent mechanistic information, and are not linked to individual electrophysiological outcomes.

We previously demonstrated that α5IA can be used as a probe drug to interrogate the HTC and effectively identify prodromal memory dysfunction using a within-subject paradigm in a transgenic model of AD. α5IA increases peak ripple amplitude in adult Fischer 344 and Long Evans male rats with wild-type cognitive function. However, the ripple band becomes unresponsive to α5IA in TgF344-AD rats by 9 months of age. These findings are consistent with the known central role of α5GABAA receptors in memory and suggest that the non-responsivity of the ripple band in TgF344-AD rats is indicative of HTC dysfunction [31, 32]. This interesting similarity emphasizes the importance of differentiating the effects of hypertension and aging from those of genetic models of hypertension and AD. It also highlights the potential utility of α5IA probe challenge as a non-invasive method to assess hippocampal network integrity in animal models for hypertension and Alzheimer’s disease as related disorders that affect memory (ADRD).

10) We assessed blood pressure by the invasive approach of femoral artery cannulation in certain studies and the non-invasive approach of tail-cuff in chronically cannulated SD rats due to technical limitations (maintaining stability of instrumented head stages). However, both approaches show elevated BP in aged males consistent with our prior reports that BP increases in aged male rats whether assessed by acute femoral artery cannulation and radiotelemetry or tail-cuff. Given the consistency of our data with prior findings, we acknowledge the limitations of BP assessment via tail-cuff but do not believe it negatively impacts the conclusions drawn from the findings reported here.

### Future directions

Based on these findings, we hypothesize that elevated MAP may disrupt HTC functional integrity independently of genetic risk factors. This offers a testable mechanistic framework for exploring vascular contributions to early memory dysfunction. Future studies should investigate whether treatment of hypertension can reverse or mitigate HTC circuit impairment. In addition, the use of connectome-selective probe drugs to evoke network responses may enable non-invasive diagnostics and guide pharmacological interventions for cognitive decline.

Our combined data suggest that high-frequency oscillations in the hippocampus represent a valuable and potentially translational biomarker. Their modulation by α5IA in the context of vascular risk when combined with emerging technologies to non-invasively measure circuitry function in adult humans opens a promising avenue for early detection and treatment development in cognitive aging.

## Perspectives

### What is new?

Aged male hypertensive subjects exhibit memory impairment, but the underlying neurophysiological changes associated with these deficits have not previously been investigated. Here, we show for the first time that the functional integrity of the hippocampal trisynaptic circuit is disrupted in aged hypertensive male Sprague–Dawley rats. Probe drug challenge with α5IA is demonstrated to be effective as an orally bioavailable probe drug to interrogate the functional integrity of the HTC.

### What is relevant?

The novel evidence of impaired functional integrity of the highly species conserved HTC provides new insight into the functional changes in neural network activity underlying memory impairments associated with age-related hypertension. These data indicate that measuring HTC neural activity in preclinical models while targeting the hippocampal circuit for dose-dependent changes in function represents a potential novel objective diagnostic tool for early detection of memory deficits associated with age-related hypertension and for optimization of novel therapeutics.

### Clinical/pathophysiological implications?

Although these preclinical results are novel, the use of probe drug challenge in the diagnosis of neurological disease is not unprecedented [38]. The probe drug challenge used by Shandilya and colleagues is like that used in our previous preclinical investigations of age-related mild cognitive impairment [33]. Assessment of HTC functional integrity with probe drug challenge using non-invasive technologies such as magnetoencephalography to measure changes in ripple band activity in patients with age-related hypertension presenting with comorbid complaints of memory impairment holds the potential to improve the translation from bench to bedside of novel therapeutic interventions [38]. However much the parallel between rodent ripple band electrophysiology and emerging human neuroimaging techniques is a tempting basis for imagining translational applications, it is premature to assess whether α5IA could be used as a translational probe drug in humans. Future research focused on the pharmacology of aging and hypertension in memory function and dysfunction would be expected to provide greater clarity of utility.

## Supplementary Information

Below is the link to the electronic supplementary material.

ESM 1 (JPEG 11.3 MB)

(PNG)

ESM 2 High Resolution

(PNG)

ESM 3 High Resolution

ESM 4 (JPEG 383 KB)

ESM 5 (JPEG 582 KB)

ESM 6 (JPEG 180 KB)

ESM 7 (DOCX 10.1 MB)

## Data Availability

All data whether primary or calculated will be available on request and will be deposited into an open access data bank.
