# Supplementary figures and images for: Age and associated hypertension impair hippocampal circuitry function and memory
Source: GeroScience. 2025 Oct 21;48(2):1571–87. doi: 10.1007/s11357-025-01831-2 (PMC12972159; doi:10.1007/s11357-025-01831-2)

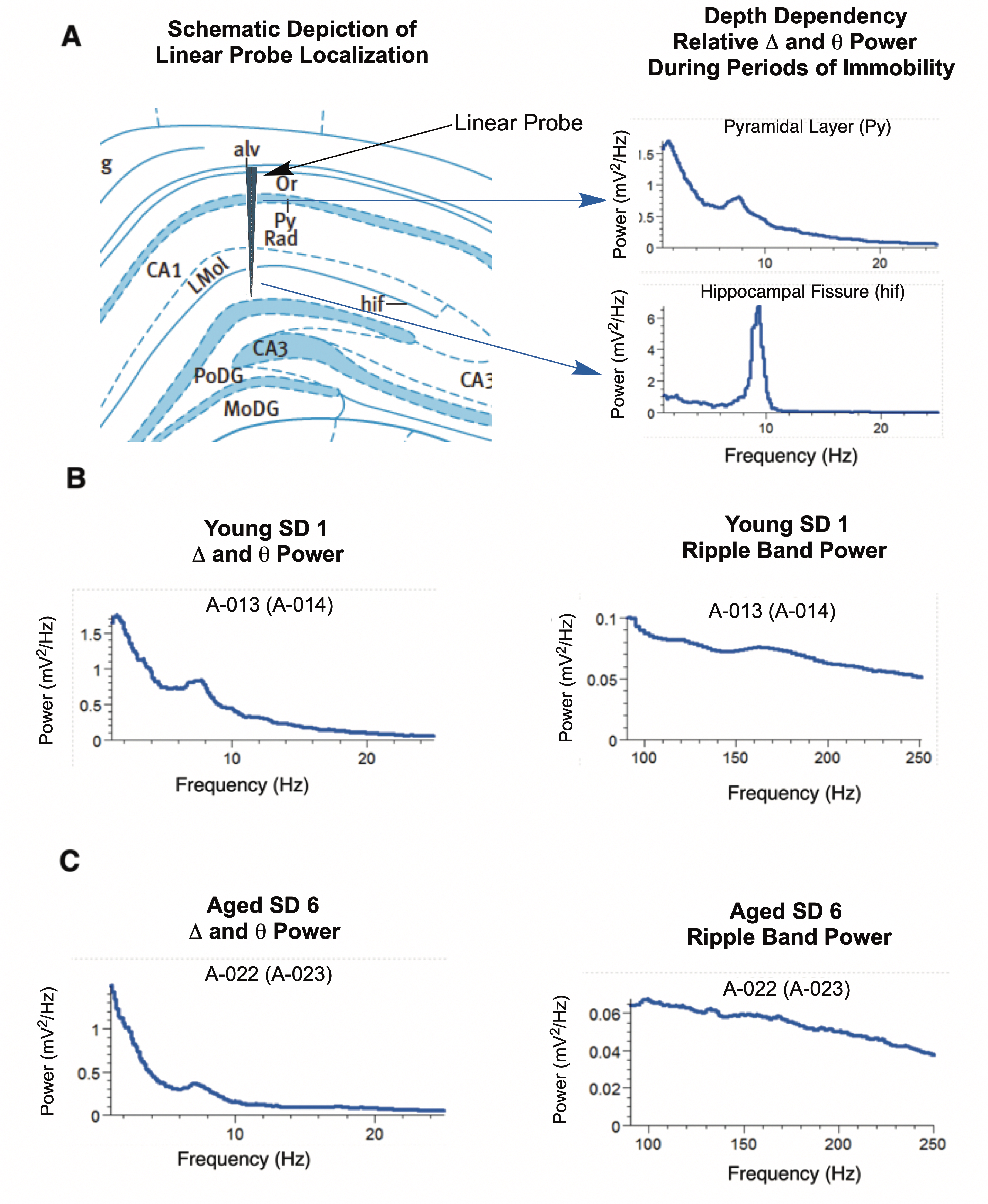

Supplement: Supplementary file 1 — (JPEG 11.3 MB) [file 11357_2025_1831_MOESM1_ESM.jpeg]

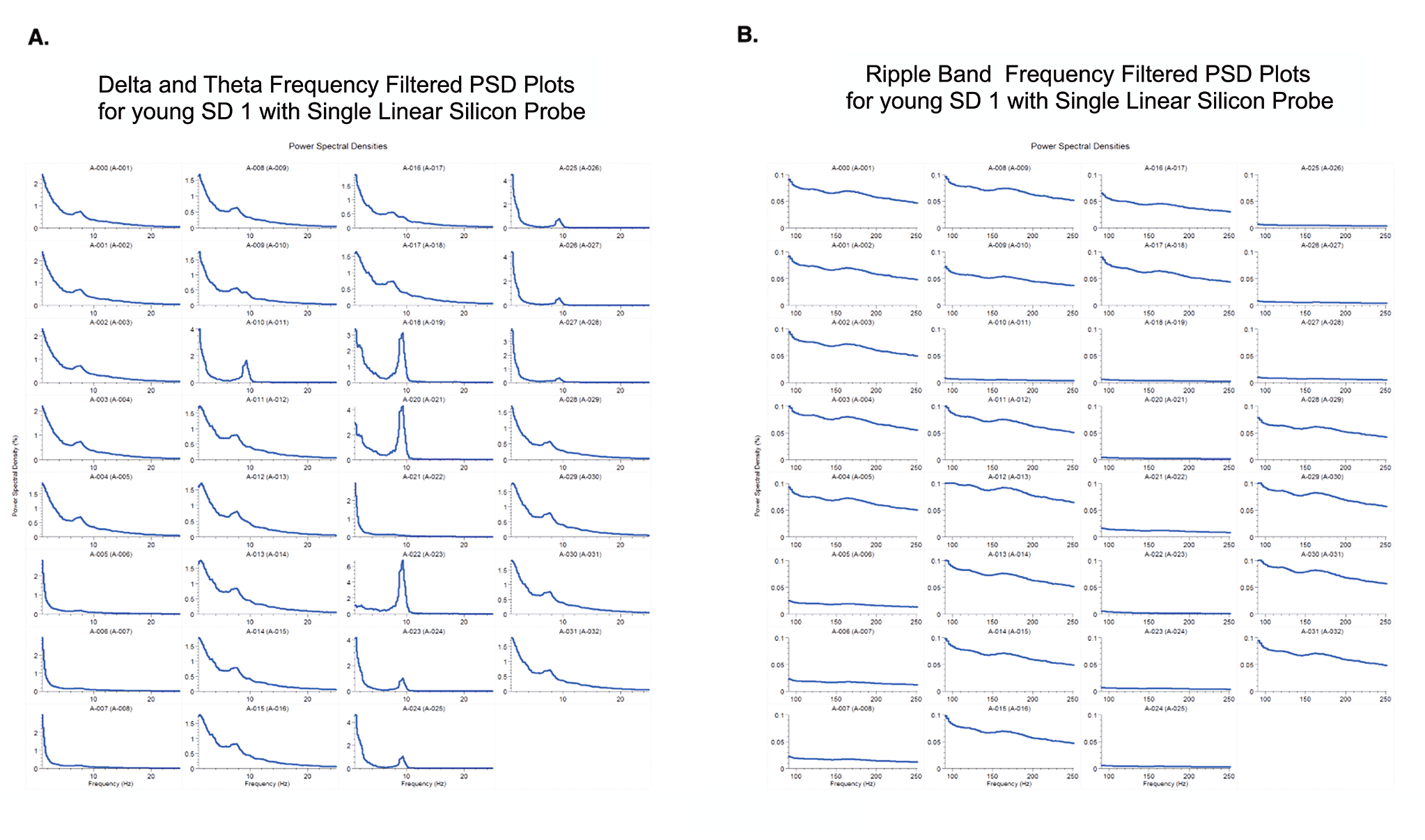

Supplement: Supplementary file 2 — (PNG) [file 11357_2025_1831_Fig7_ESM.png]

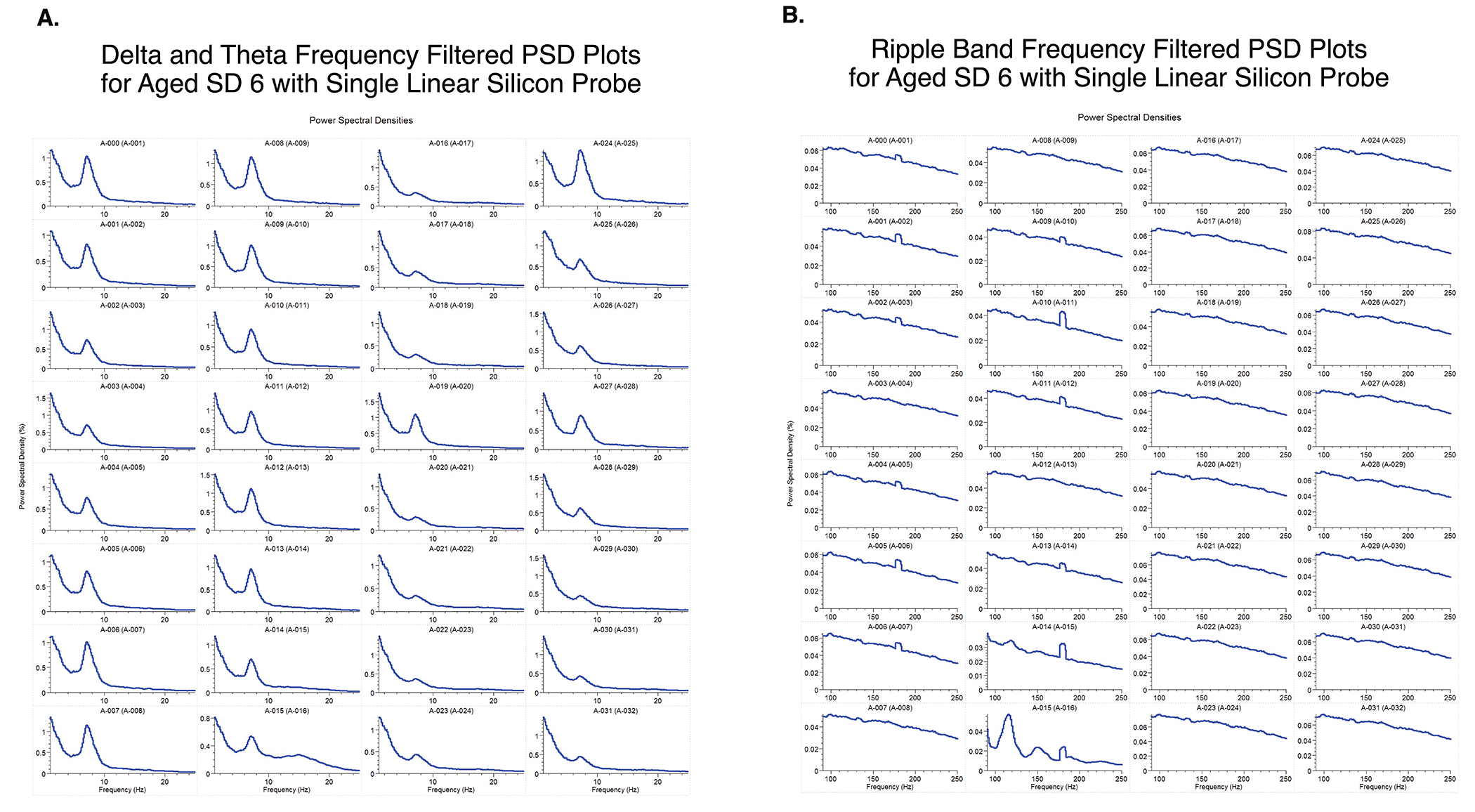

Supplement: Supplementary file 4 — (PNG) [file 11357_2025_1831_Fig8_ESM.png]

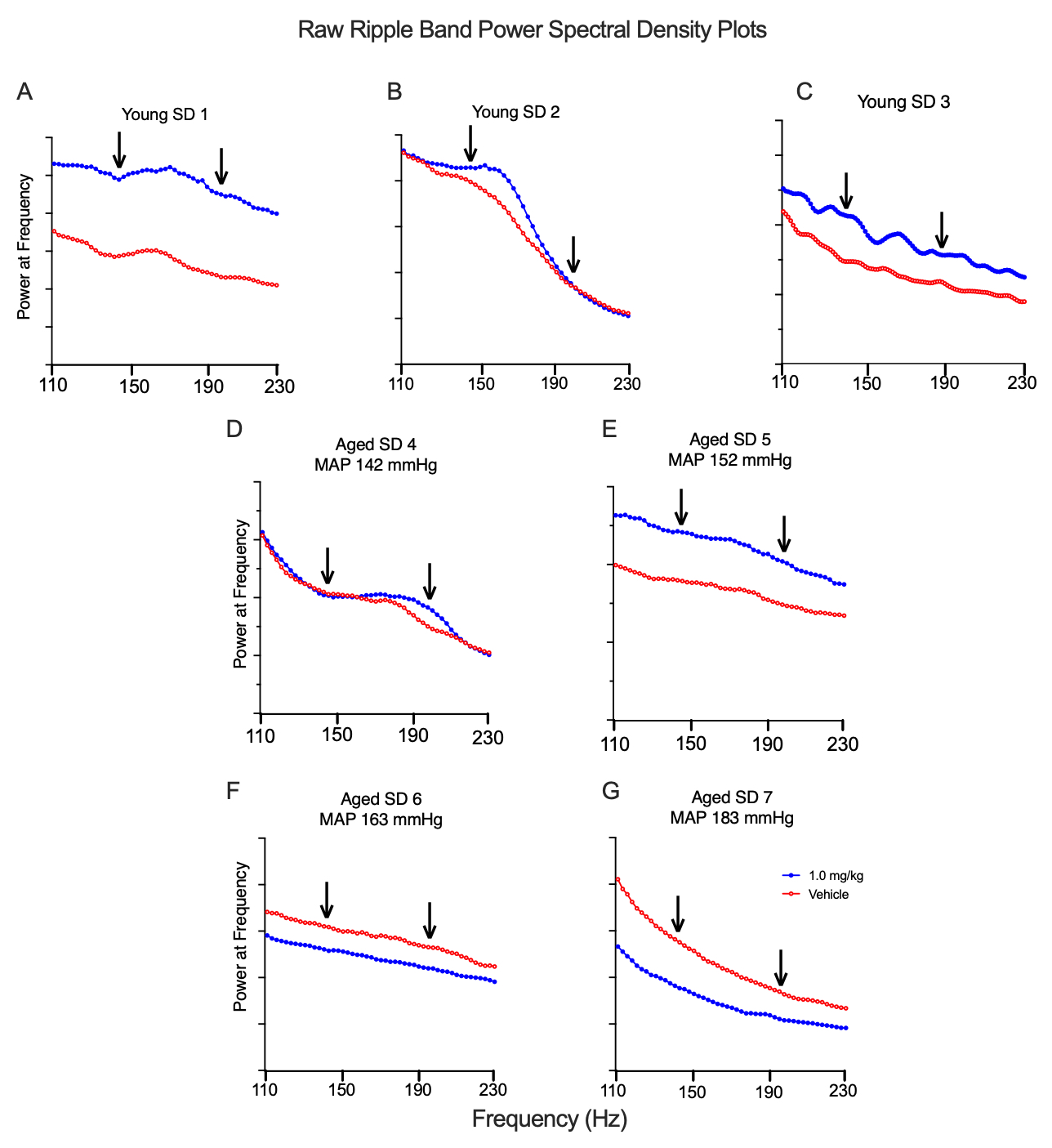

Supplement: Supplementary file 6 — (JPEG 383 KB) [file 11357_2025_1831_MOESM4_ESM.jpeg]

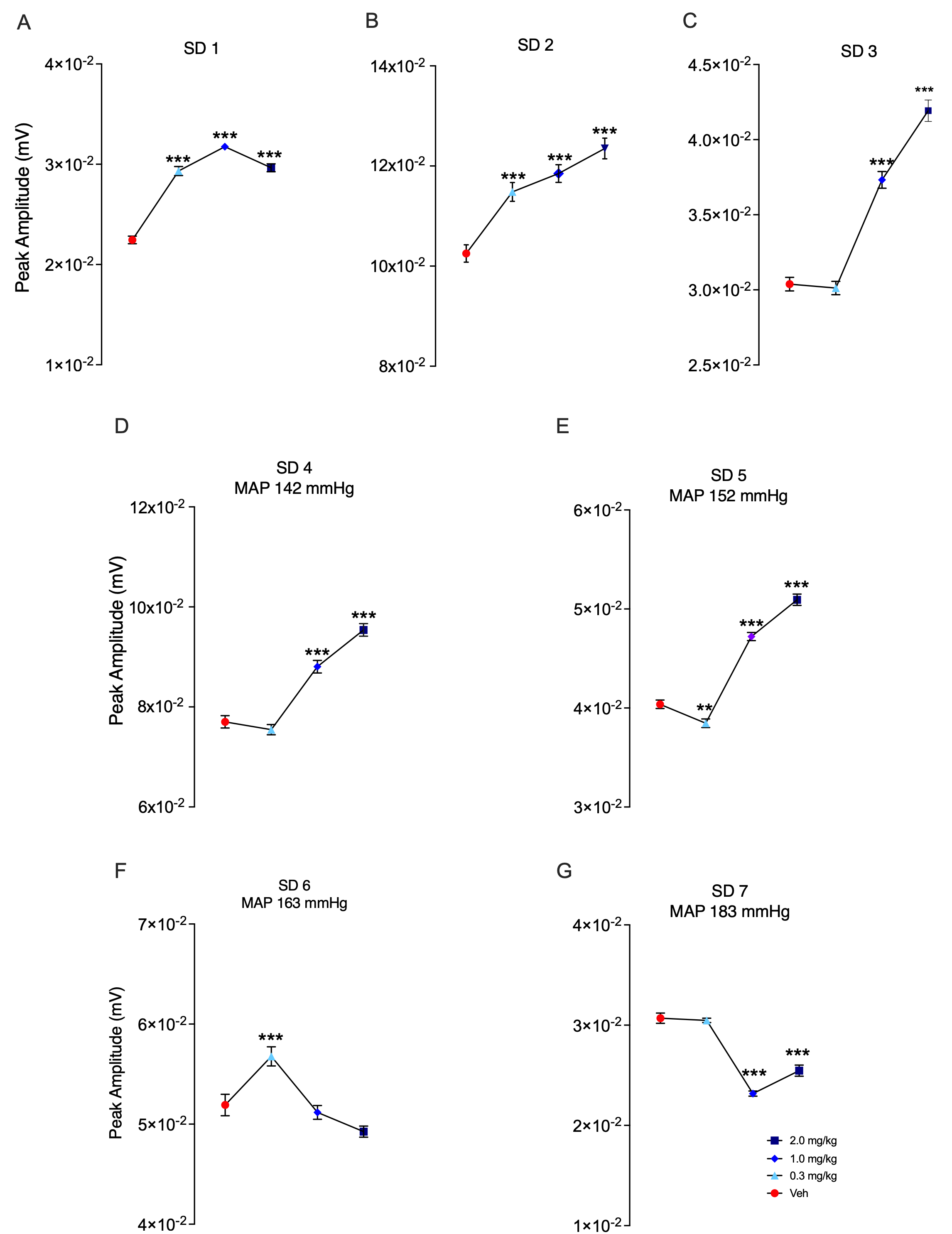

Supplement: Supplementary file 7 — (JPEG 582 KB) [file 11357_2025_1831_MOESM5_ESM.jpeg]

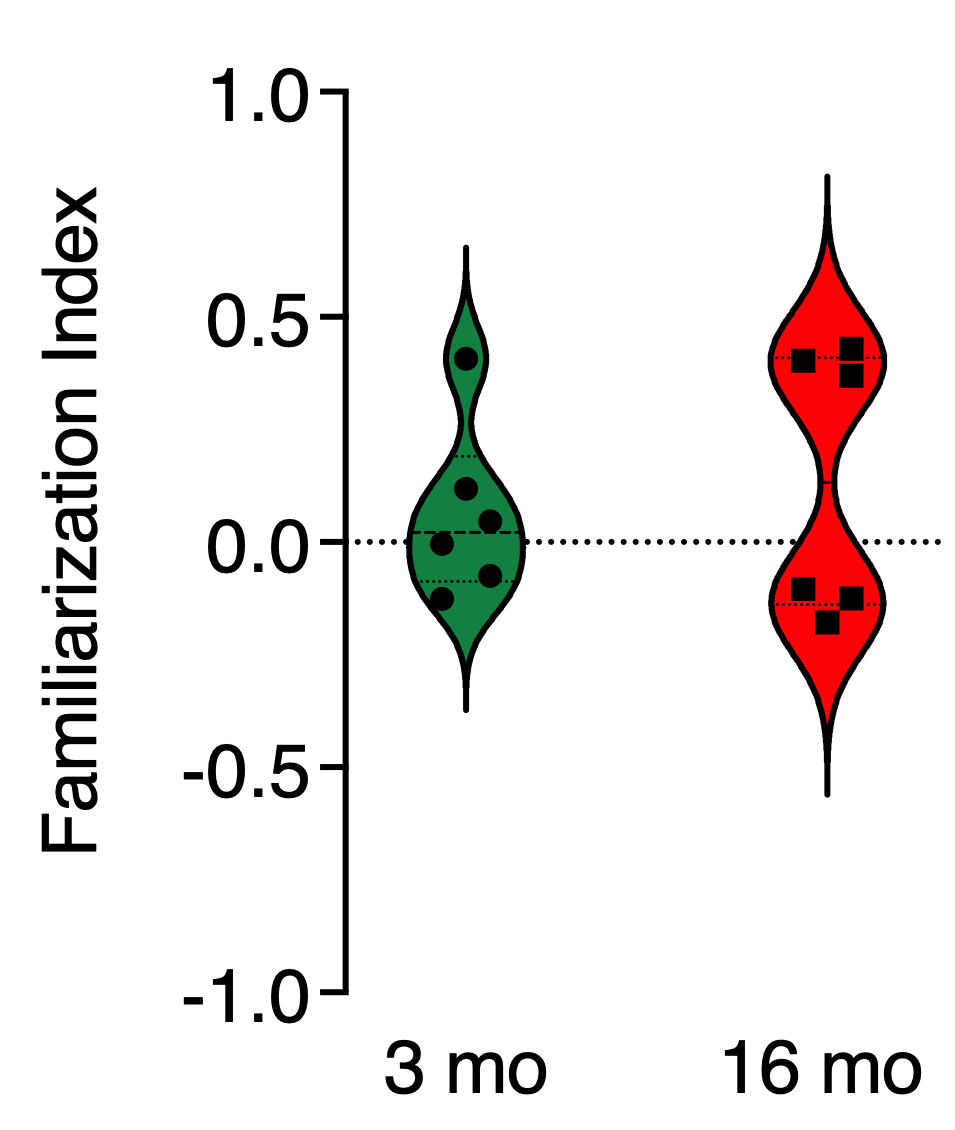

Supplement: Supplementary file 8 — (JPEG 180 KB) [file 11357_2025_1831_MOESM6_ESM.jpeg]
