# Supplementary material for: Age and associated hypertension impair hippocampal circuitry function and memory
Source: GeroScience. 2025 Oct 21;48(2):1571–87. doi: 10.1007/s11357-025-01831-2 (PMC12972159; doi:10.1007/s11357-025-01831-2)
Supplement: Supplementary file 9 — (DOCX 10.1 MB) [file 11357_2025_1831_MOESM7_ESM.docx]

***Supplemental Data for:* Age and Associated Hypertension Impairs Hippocampal Circuitry Function and Memory**

**Authors and Affiliations:**

Marcia H. Ratner^1^, Kayla Nist ^2^ Richard Wainford^1,3^ and David H. Farb ^1*^

^1^Department of Pharmacology, Physiology and Biophysics, Boston University Chobanian & Avedisian School of Medicine, Boston, Massachusetts, USA.

^2^Department of Anatomy and Neurobiology, Boston University Chobanian & Avedisian School of Medicine, Boston, Massachusetts, USA.

^1,3^Department of Medicine, Division of Cardiology, Emory University School of Medicine, Atlanta, GA, USA

*Corresponding author. Email: dfarb@bu.edu

Ratner, Marcia marcia@bu.edu

Nist, Kayla kmnist@bu.edu

Wainford, Richard David richard.david.wainford@emory.edu

Farb, David H dfarb@bu.edu

**Table S1: Baseline MAP, SBP, Age at *In Vivo* Electrophysiological Testing and Response to Probe Drug Challenge in Male SD Rats.**

| **Rat Number** | **MAP** | **SBP** | **Age at time of**  ***In Vivo* Electrophysiological Testing**  **(Months/Days)** | **Response to** a**5IA Probe**  **Drug Challenge** |
| --- | --- | --- | --- | --- |
| Young SD (Rat 1)* | N/R | N/R | 5/0 | Positive Responder |
| Young SD (Rat 2) | N/R | N/R | 3/17 | Positive Responder |
| Young SD (Rat 3) | N/R | N/R | 5/1 | Positive Responder |
| Aged SD (Rat 4) | 142 | 153 | 14/26 | Positive Responder |
| Aged SD (Rat 5) | 152 | 159 | 15/15 | Positive Responder |
| Aged SD (Rat 6)* | 163 | 174 | 16/19 | ***Non-responder*** |
| Aged SD (Rat 7) | 183 | 191 | 16/29 | ***Non-responder*** |

N/R = not recorded; MAP = Mean Arterial Blood Pressure; SBP = Systolic Blood Pressure; * linear probe


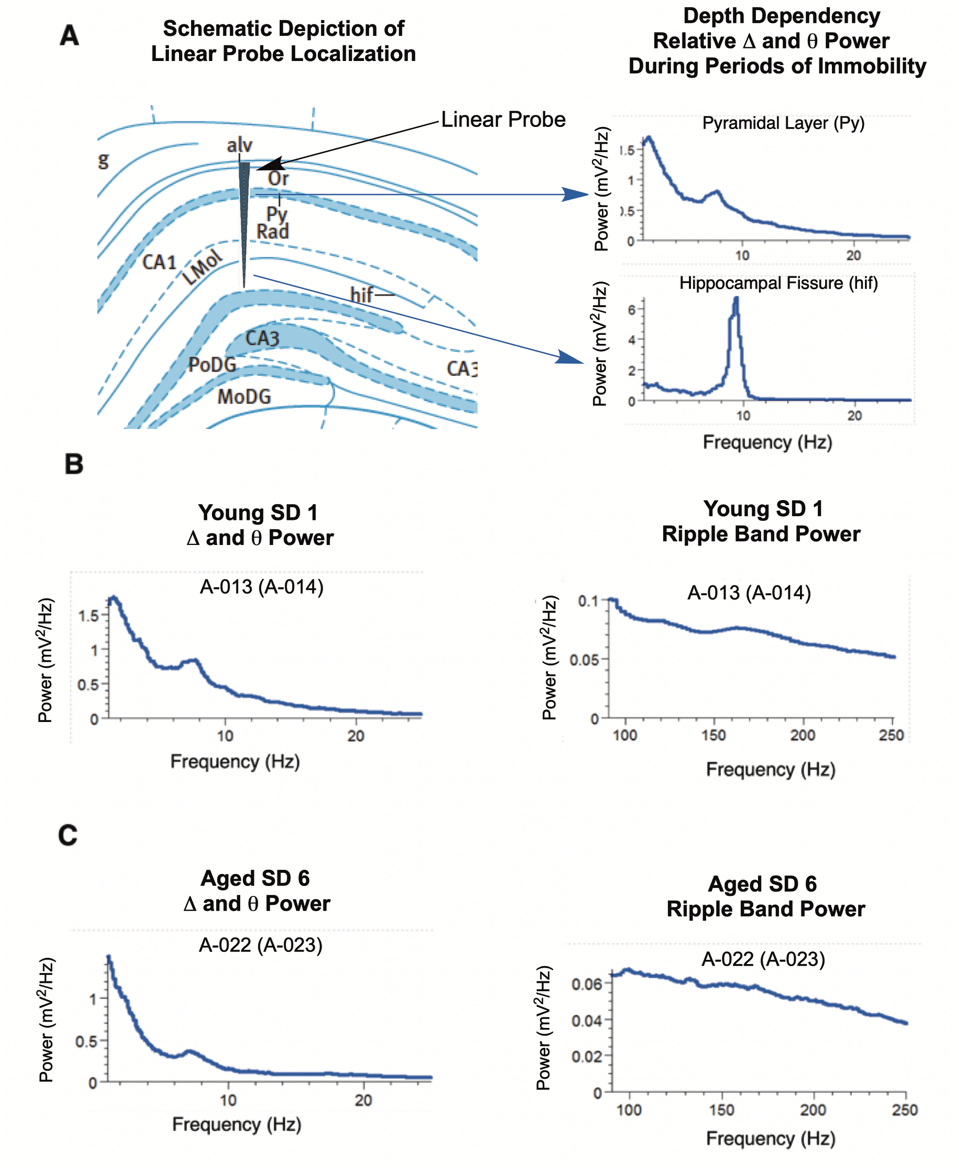


**Figure S1:** Power spectral density (PSD) plots for a **young (SD 1)** and an **aged (SD 6)** subject each implanted with linear single shank silicon probe comprising 32 probes aligned along its length. **A) Young (SD 1):** PSD plots showing delta and theta frequency ranges illustrates how delta (Δ, 0.5 to 4 Hz) and theta (Θ, 4 to 12 Hz) power depends on electrode depth: Top panel electrode A-012 (A-013) in pyramidal layer (Py) vs bottom panel electrode A-022 (A-023) in the hippocampal fissure (hif). Maximum theta power and minimal delta is seen in an electrode closer to the hippocampal fissure.  **B)** **Young (SD 1):** left panel, delta and theta power as in A but from a nearby electrode (A-013 (A-014)) shows a similar PSD plot with high delta and low theta consistent with our experience that proximal electrodes in CA1 show very similar but not identical PSD spectra. Here, theta power is low relative to delta power consistent with the lack of ambulation as required by the awake immobility model for uncovering sharp wave ripple activity.  Right panel shows LFP data from 90 to 250 Hz. Note the characteristic hump in the ripple band from 140 to 200 Hz. **C)** **Aged (SD 6):** PSD Plots shows relative flattening of the PSD spectra and absence of hump, characteristic in the ripple band in normative subjects, from 140 to 200 Hz.

The raw PSD plots from the vehicle sessions of these animals are shown for comparison **(see Figs: S2 & S3).**  The plots from the young SD Rat 1 show overt depth-dependent qualitative and quantitative differences in theta and delta power ratios on different electrodes as indicative of electrode location in CA1, stratum radiatum or stratum oriens.  Many electrodes also show an overt ripple hump from 140 to 200 Hz when the LFP data is frequency filtered for 90 to 250 Hz.  By contrast, none of the electrodes from aged SD 6 show an overt ripple band hump despite also showing depth-dependent qualitative and quantitative differences in theta and delta power ratios on different electrodes. This suggests that there is an occult story here yet to be resolved concerning the impact of aging and/or hypertension dynamic on synchronous activity. As with young SD Rat 1 many electrodes showed an overt ripple hump from 140 to 200 Hz when the LFP data was frequency filtered at 90 to 250 Hz.  This indicates that the lack of a response to probe drug in aged SD 6 and the other aged SDs with high MAP is unlikely due to differences in probe placement within CA1, particularly considering the density of surrounding electrodes that yield internally consistent results.    The raw PSD ripple band data plots from all rats are shown in Figure S4.


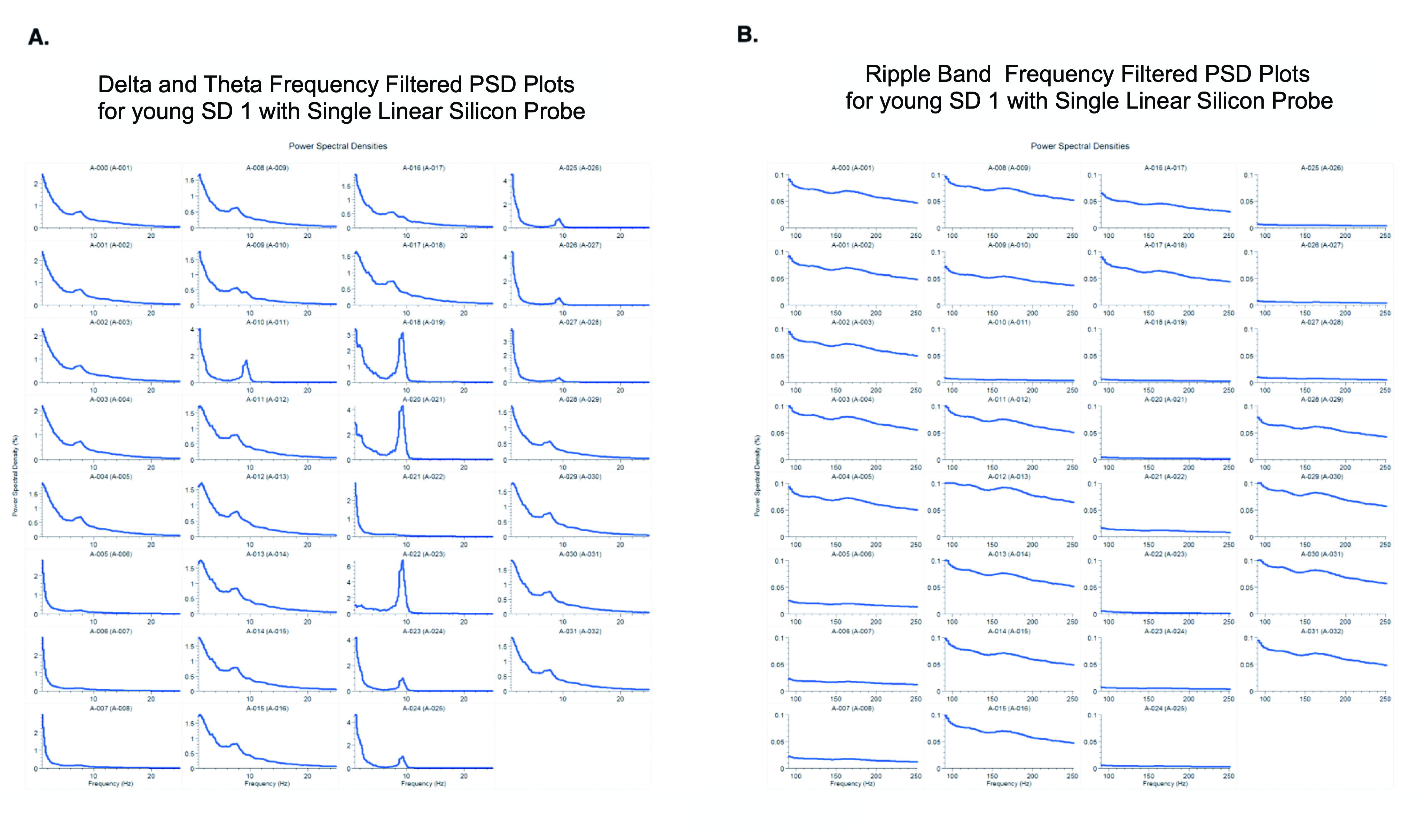


**Figure S2:** PSD Plots for young male SD Rat 1 implanted with a linear single shank silicon probe. **A)** Panel shows depth dependency for LFP signal power in the theta and delta frequency bands.  **B)** Panel shows depth dependency for baseline LFP signal power in the ripple band.


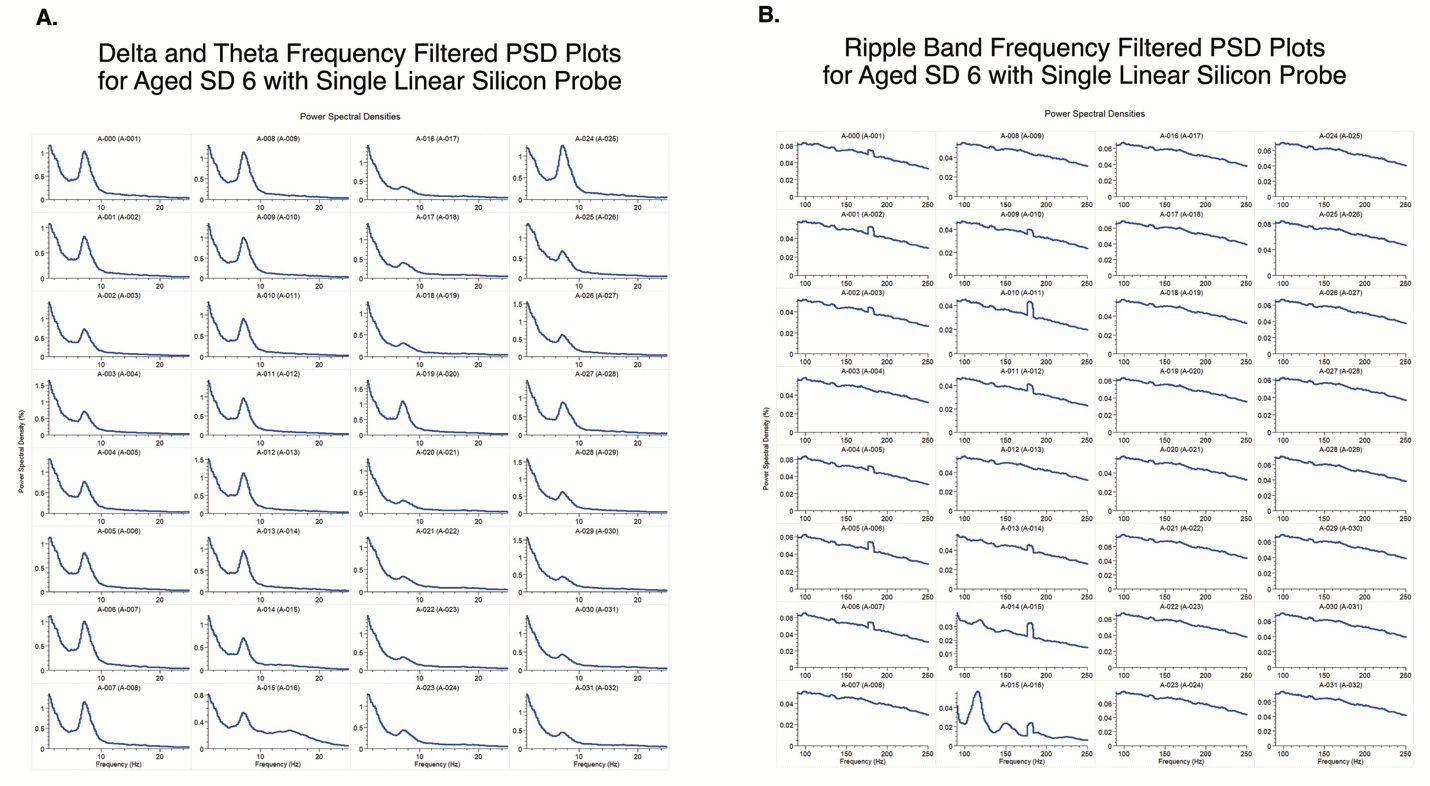


**Figure S3:** PSD Plots for aged SD Rat 6 implanted with a linear single shank silicon probe**. A)** Panel showing depth dependency for LFP signal power in the theta and delta frequency bands. **B)** Panels show depth dependency for baseline LFP signal power in the ripple band.


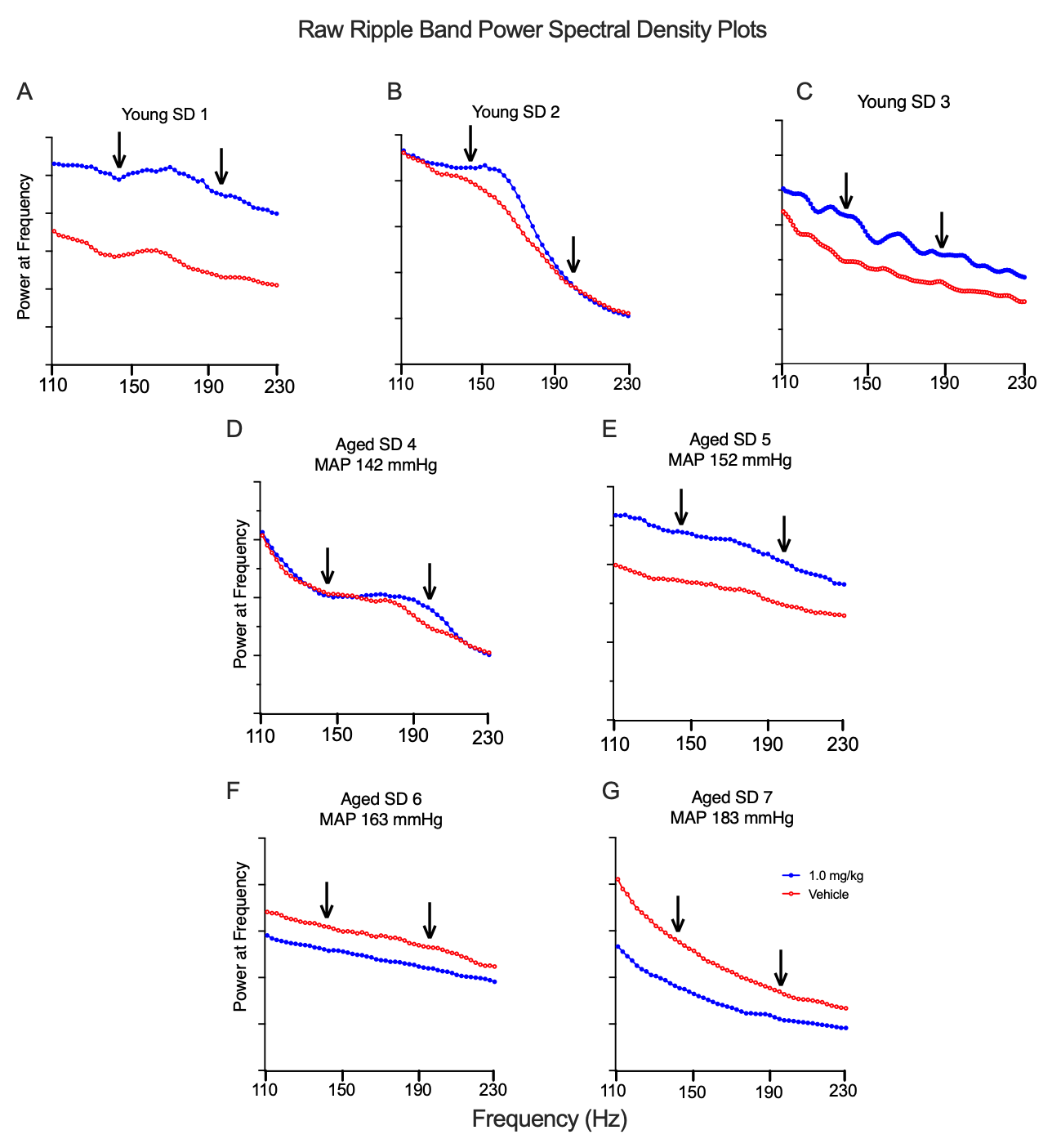


**Figure S4:** **Raw** **Power Spectral Density (PSD) Plots for all Rats**.  PSD plots show relative frequency filtered local field potential power in mV^2^/Hz on the ordinate axis with frequencies in Hz on the abscissa following administration of vehicle and 1.0 mg/kg of α5IA probe drug**.**Vertical black arrows indicate beginning and end of the ripple band.  **(A & B)** The PSD plots of young male SD rats (n = 2) show an overt hump in the ripple band indicated by arrows and an increase in relative power over vehicle baseline following administration of α5IA probe drug.  (**C)** One young SD does not show the usual ripple band hump but nevertheless shows an increase in band power relative to vehicle (red line). (**D)** PSD plot of aged SD (Rat 4) with MAP of 142 mmHg also shows an overt hump in the ripple band that increases following administration of the 1.0 mg/kg dose of α5IA but the frequency distribution is shifted to right.  (**D)** PSD plot of aged SD (Rat 5) which had the second lowest MAP also shows an overt increase in ripple band power in response to probe drug challenge but, the characteristic hump in the ripple band is overtly attenuated.  (**E-F)** PSD plots from the two aged SDs (Rats 6 and 7) with severe hypertension associated with MAPs greater than 160 mmHg reveals a conspicuous absence of the ripple band hump and a loss of the increase in ripple band power typically seen in response to 5IA probe drug challenge.  Key shown in Panel G

**
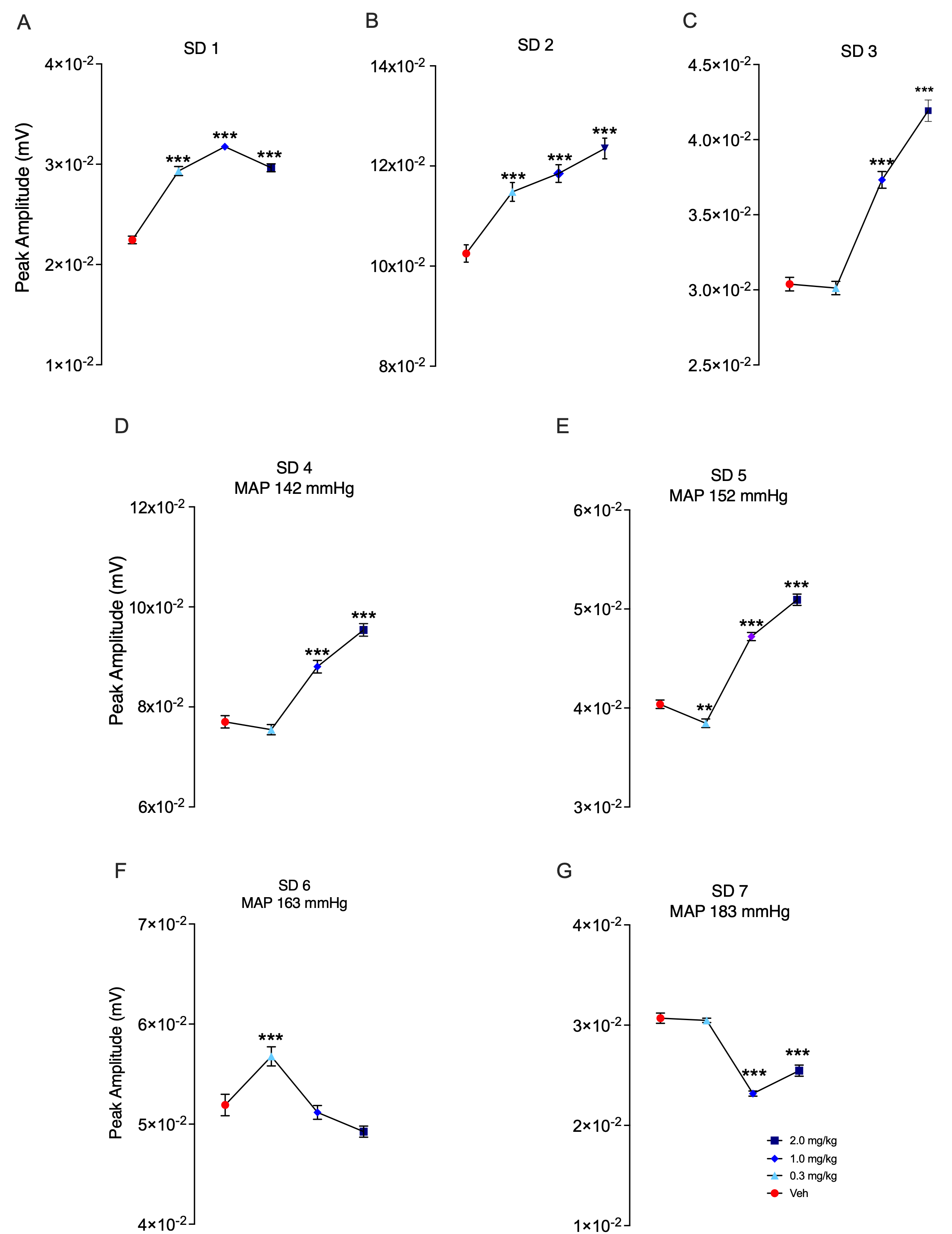
**

**Figure S5.** **Peak Ripple Amplitudes of SD Rats**.  Plots show significant within subject changes in peak ripple amplitudes following administration of escalating doses of α5IA probe drug in young and aged SD rats**. A-C)** Peak ripple amplitudes of young SDs showing significant within subject increase following administration of the 1.0 and 2.0 mg/kg doses of α5IA.  **D-E)** Peak ripple amplitudes of aged SDs with MAPs <160 mmHg also showing significant increases following administration of the 1.0 and 2.0 mg/kg doses of α5IA. **F-G)** Peak ripple amplitudes of aged SDs with MAPs >160 mmHg do not show the expected significant increases following administration of the 1.0 and 2.0 mg/kg dose of α5IA.  Key shown in Panel G.

**Figure S6**: Familiarization index scores showing no significant difference (p = 0.63) between two age groups for time spent exploring the objects used in the location and object recognition experiments.
